# Hepatitis B virus rigs the cellular metabolome to avoid innate immune recognition

**DOI:** 10.1038/s41467-020-20316-8

**Published:** 2021-01-04

**Authors:** Li Zhou, Rui He, Peining Fang, Mengqi Li, Haisheng Yu, Qiming Wang, Yi Yu, Fubing Wang, Yi Zhang, Aidong Chen, Nanfang Peng, Yong Lin, Rui Zhang, Mirko Trilling, Ruth Broering, Mengji Lu, Ying Zhu, Shi Liu

**Affiliations:** 1 State Key Laboratory of Virology, Modern Virology Research Center, College of Life Sciences, Wuhan University, Wuhan, 430072 China; 2 College of Bioscience and Biotechnology, Hunan Agricultural University, Changsha, 410128 China; 3 The Key Laboratory of Biosystems Homeostasis and Protection of the Ministry of Education and Innovation Center for Cell Signaling Network, Life Sciences Institute, Zhejiang University, Hangzhou, China; 4 Department of Laboratory Medicine, Zhongnan Hospital of Wuhan University, Wuhan, China; 5 Hubei Provincial Cooperative Innovation Center of Industrial Fermentation, College of Food and Pharmaceutical Engineering, Hubei University of Technology, Wuhan, 430068 China; 6 Department of Physiology, Nanjing Medical University, Nanjing, 211166 China; 7 The Key Laboratory of Molecular Biology of Infectious Diseases designated by the Chinese Ministry of Education, Chongqing Medical University, Chongqing, China; 8 Department of Hepato-Pancreato-Biliary Surgery, SunYat-sen Memorial Hospital, SunYat-sen University, Guangzhou, 510120 China; 9 Institute for Virology, University Hospital Essen, University of Duisburg-Essen, Essen, 45122 Germany; 10 Department of Gastroenterology and Hepatology, University Hospital Essen, University of Duisburg-Essen, Essen, 45122 Germany

**Keywords:** Infection, Hepatitis B virus, Viral hepatitis

## Abstract

Glucose metabolism and innate immunity evolved side-by-side. It is unclear if and how the two systems interact with each other during hepatitis B virus (HBV) infections and, if so, which mechanisms are involved. Here, we report that HBV activates glycolysis to impede retinoic acid-inducible gene I (RIG-I)-induced interferon production. We demonstrate that HBV sequesters MAVS from RIG-I by forming a ternary complex including hexokinase (HK). Using a series of pharmacological and genetic approaches, we provide in vitro and in vivo evidence indicating that HBV suppresses RLR signaling via lactate dehydrogenase-A-dependent lactate production. Lactate directly binds MAVS preventing its aggregation and mitochondrial localization during HBV infection. Therefore, we show that HK2 and glycolysis-derived lactate have important functions in the immune escape of HBV and that energy metabolism regulates innate immunity during HBV infection.

## Abstract (web summary)

RIG-I is a cytosolic antiviral nucleic acid sensor that signals via MAVS to produce type 1 interferons. Here the authors show that hepatits B virus can repress this pathway by activating glycolysis and lactate production, enabling accumulated lactate to bind MAVS and prevent its mitochondrial localization.

## Introduction

The innate immune system recognizes pathogens via pattern recognition receptors such as Toll-like receptors (TLR) and retinoic acid-inducible gene I (RIG-I)-like receptors (RLRs)^1,2^. RLRs, including RIG-I and MDA5, are cytosolic RNA sensors that trigger innate immune responses by recruiting a signaling adaptor called mitochondrial antiviral-signaling (MAVS) (also known as Cardif, VISA, and IPS-1)^3,4^. Once activated, MAVS molecules form prion-like supra-molecular aggregates at mitochondria, which recruit and activate TBK1 and IKKε^5,6^. Activated TBK1 and IKKε induce the activation of transcription factors, including IRF3 and nuclear factor κB, leading to the production of type I interferon (IFN) and pro-inflammatory cytokines^7^. Secreted type I IFNs (IFN-I) bind to the cognate IFNα/β receptor (IFNAR) being present on the surface of most nucleated cells. The IFNAR receptor initiates Jak/STAT signaling, resulting in the transcription of IFN-stimulated genes (ISGs). ISGs fulfill various biological functions executing antiviral activity^8^.

Upon viral infections, immune cells increase their glucose uptake and utilization to meet their demand for energy and molecular building blocks^9,10^. After internalization through the glucose transporter, glucose molecules are either processed by glycolysis, the hexosamine biosynthesis pathway (HBP) or the pentose phosphate pathway^11^. Each pathway has distinct functions and purposes. During glycolysis, glucose is metabolized to pyruvate, which can be further metabolized by two different ways: (I) in presence of oxygen, pyruvate is processed by the pyruvate dehydrogenase complex (PDHc) in mitochondria through the tricarboxylic acid cycle (TCA), leading to the generation of acetyl-CoA^12^. (II) In absence of oxygen, lactate dehydrogenase (LDH) uses pyruvate to generate lactate^13^. In the past, lactate was often considered as useless metabolic end product. Recently, however, several studies demonstrate the importance of lactate concerning the regulation of various cellular processes, which are implicated in tumor immune surveillance, T-helper cell differentiation, and macrophage polarization^9,10^.

Hepatitis B virus (HBV) is a partially double-stranded DNA (dsDNA) virus that encodes several viral proteins such as the DNA polymerase, the surface antigen (HBsAg), the core antigen (HBcAg), and the X protein (HBx)^14^. The HBV life cycle is special. After entry into cells, the dsDNA genome is transported to the nucleus. The partially double-stranded DNA genome is repaired forming a covalently closed circular DNA (cccDNA) genome^15,16^. The nuclear cccDNA serves as a template for pregenomic RNAs (pgRNA) and subgenomic RNA transcripts, encoding all viral proteins^15,16^. RIG-I recognizes the pgRNA of HBV and activates innate immunity^17^.

Recently, a landmark study shows that lactate is a natural suppressor of RLR signaling by targeting MAVS^18^. Here, we examine this model and its exploitation by HBV. We demonstrate a novel mechanism of HBV-mediated immune escape through which HBV attenuates RLR signaling using glycolysis. Mechanistically, the HBV-induced lactate binds MAVS, leading to impaired mitochondrial localization of MAVS competing with the interaction of RIG-I and MAVS, and impairing MAVS aggregation, which is necessary for its function in stimulating innate immunity.

## Results

### HBV inhibits RLR signaling by promoting glucose metabolism

**Fig. 1:**
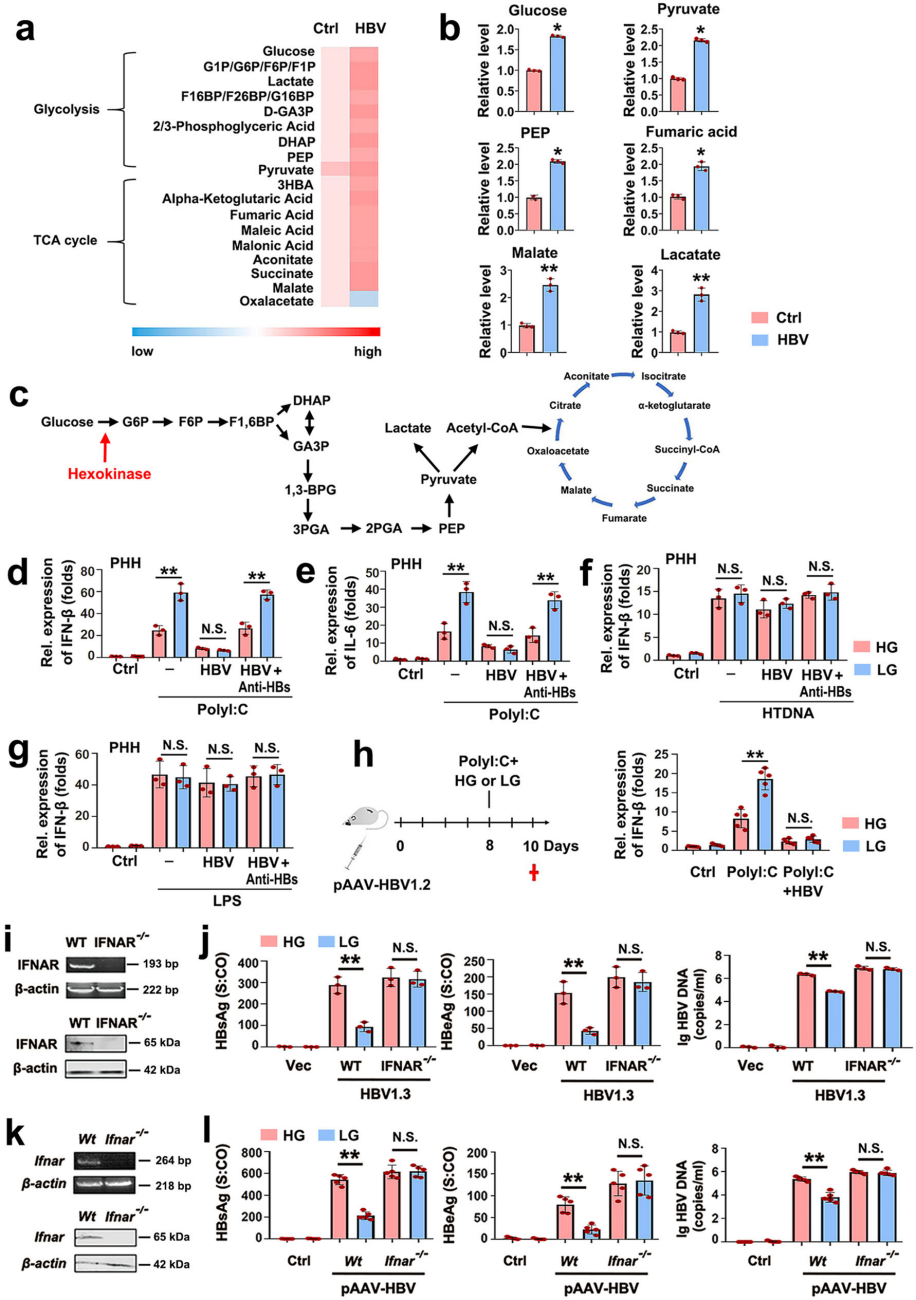
HBV inhibits RLR signaling by promoting glucose metabolism. **a** PHHs were infected with or without HBV virions (MOI = 30) for 9 days and harvested for metabolomics analysis. A heat map shows changes of glycolysis or oxidative phosphorylation metabolites. **b** Quantification of indicated intermediates of glycolysis and TCA cycle. **c** Simplified scheme of the glucose metabolic pathway. **d**, **e** PHHs were infected with or without HBV virions (MOI = 30) for 7 days. The supernatants were pretreated with or without anti-HBs, an HBV neutralizing antibody. Then, cells were cultured with high glucose (25 mM) or low glucose (5 mM) for 36 h, and treated with or without poly(I:C) (1 μg/mL) for 12 h prior to qPCR analyses. **f**, **g** Experiments were performed similar to those in **d**, **e**, except that cells were treated with or without HTDNA (1 μg/mL) or LPS (50 ng/mL). **h** C57BL/6 mice received HI with 10 µg of plasmid pAAV-HBV1.2 for 7 days. Mice were fasted overnight and then treated with high glucose (1.5 g/kg) or low glucose (0.2 g/kg) and were treated with PBS or poly(I:C) (10 μg/g) for 2 days (left panel). Mouse liver samples were collected and subjected to qPCR analyses (right panel). **i** IRNAR1 expression in IFNAR1 WT (WT) and IFNAR1−/− cells was measured using RT-PCR and western blot analyses. **j** IFNAR1 WT (WT) and IFNAR1−/− cells were cultured with high glucose or low glucose and transfected with pHBV-1.3 for 48 h. HBsAg and HBeAg (middle panel) secreted in culture supernatants were quantified by chemiluminescence immunoassay (CMIA), or HBV genomes in culture supernatants were determined by qPCR. **k** IFNAR expression in liver (*n* = 3) was measured using RT-PCR and western blot analyses. **l** Experiments were performed similar to those in **h**, except HBsAg, HBeAg and HBV DNA were analyzed. Data **b**, **d**–**g**, **j** represent the means ± SD (Student’s *t* test), data **h**, **j**, **l** represent the means ± SEM (one-way ANOVA) (**P < 0.01; *P < 0.05, n.s., not significant). *S:CO* signal to cutoff ratio, *HG* high glucose, *LG* low glucose, *HI* hydrodynamic injection. See also Supplementary Figs. 1 and 2.

To investigate global metabolic changes during HBV infection, we applied an unbiased systemic approach. We found that the abundance of most metabolic intermediates downstream of glucose was elevated during HBV infection, including phosphoenolpyruvate, pyruvate, and lactate (Fig. 1a–c). To assess the biological relevance of this metabolic deregulation, primary human hepatocytes (PHHs) were infected with HBV and incubated with high or low glucose levels. As determined by quantitative real-time reverse-transcription polymerase chain reaction (qPCR), PHHs treated with low glucose media showed much stronger induction of IFN-β and interleukin-6 (IL-6) mRNA upon poly(I:C) transfection compared with cells under high glucose treatment (Fig. 1d, 1e). Conversely, the HBV infection inhibited IFN-β and IL-6 production irrespective of the glucose concentrations (Fig. 1d, 1e). This HBV-mediated inhibition was sensitive to human HBV-specific immunoglobulin preparations containing neutralizing antibodies that prevent the infection (Fig. 1d, e). Similar results were observed in the well-established HepG2-NTCP HBV infection cell system as well as stably HBV-infected HepG2.2.15 cells (Supplementary Fig. 1a–c). The efficacy of anti-HBV IgG for HBV neutralization was ensured by qPCR detecting HBV-DNA (Supplementary Fig. 1d). The HBV infection inhibited TBK1-IRF3 signaling and RLR/RIG-I-responsive gene expression, induced by poly(I:C), including Mx1 and RSAD2 (Supplementary Fig. 1e, f). Conversely, the IFN induction following treatment with herring testis DNA (HTDNA) or lipopolysaccharide that activate STING and TLR signaling, respectively, was not affected by glucose (Fig. 1f, g and Supplementary Fig. 1g). To test the relevance of the glucose catabolism for HBV immune escape in vivo, an HBV hydrodynamic injection (HI) mouse model was established in C57BL/6 mice. As shown in Fig. 1h, high glucose concentrations inhibited the IFN-β production upon poly(I:C) injection. We then compared the influence of four HBV genotypes (A-D) on glucose-regulated IFN-β expression. The experiments indicated that all four HBV genotypes suppressed IFN-β expression upon poly(I:C) transfection (Supplementary Fig. 1h). To test whether the glucose catabolism regulates HBV replication through IFN, we generated IFNAR1-deficient Huh7 cells (IFNAR^−/−^ cells) using CRISPR/Cas9 technology and confirmed the absence of IFNAR1 (Fig. 1i). As expected, glucose deprivation diminished the levels of secreted HBsAg, HBeAg, and HBV-DNA in the serum of WT cells, but did not affect HBV replication in IFNAR1-deficient cells (Fig. 1j). To further explore whether the glucose catabolism regulates HBV replication through IFN in vivo, we employed the *Ifnar*1 knockout (KO) (*Ifnar1*^*−/−*^) mouse model and confirmed IFNAR1 deficiency (Fig. 1k). In agreement with the in vitro assays, we found that glucose deprivation inhibits serum HBsAg, HBeAg, and HBV-DNA in WT mice but not in *Ifnar*^*−/−*^ mice (Fig. 1l).

Because the low-glucose condition still affects HBV replication even though HBV blocks IFN responses to poly(I:C) in both the high- and low-glucose condition (Fig. 1j), we hypothesized that poly(I:C)-induced signaling is not the only pathway involved in glucose-regulated HBV replication. In our previous study, we demonstrated that glucosamine increases HBV replication by regulating the autophagic machinery^19^. Therefore, we asked whether glucose also modulates HBV replication through autophagic flux. As shown in Supplementary Fig. 2a, the number of MAP1LC3/LC3 (microtubule-associated protein 1 light chain 3) puncta was increased in response to high-glucose treatment in HepG2 cells. Similarly, high glucose treatment induced the number of LC3 puncta and HBsAg expression. (Supplementary Fig. 2b). Of note, LC3 puncta and HBsAg colocalized in HBV-transfected HepG2 cells (Supplementary Fig. 2b). This phenomenon was consistent with a published finding^20^. In addition, western blot analysis revealed that high glucose treatment enhanced the levels of the autophagic cargos LC3-II and SQSTM1, as well as HBcAg expression (Supplementary Fig. 2c). In a previous study, chloroquine (CQ) treatment led to the inhibition of cargo degradation and lysosomal compartment acidification in lysosomes^21^. Like CQ treatment, an accumulation of LC3 puncta with strong expression of both GFP and mCherry was observed in high glucose treated cells (Supplementary Fig. 2d). The results indicated that high-glucose treatment led to incompleted autophagy and reduced cargo degradation in autophagosomes. In accordance with those results, high-glucose treatment inhibited the fluorescent signal of dye quenched-bovine serum albumin (Supplementary Fig. 2e). Earle’s balanced salt solution treatment was included as a positive control (Supplementary Fig. 2e). We suspected that glucose prevents autophagic degradation by reducing autophagosome–lysosome fusion or regulating lysosomal proteolysis activity. To confirm our speculation, lysosomal associated membrane protein 1 (LAMP1), a lysosome marker, was employed. Using confocal microscopy, we showed that high-glucose treatment induced the colocalization of HBsAg and LAMP1 (Supplementary Fig. 2f). These results indicated that high glucose treatment could not affect autophagosome–lysosome fusion. Thus, we further assessed the role of glucose on the acidification of lysosomal compartments. As shown in Supplementary Fig. 2g, high-glucose treatment suppressed the fluorescence intensities of LysoTracker and LysoBeacon staining (Supplementary Fig. 2g). Similar results were obtained by using acridine orange (AO), which eliminates bright red fluorescence when entering acidic lysosomes (Supplementary Fig. 2h). Together, these results demonstrate that HBV induces a metabolic alteration, lead to inhibiting poly(I:C)-induced IFN-β production and suppressing autophagic degradation.

### HBV controls glucose and IFN signaling via a ternary complex

We further investigated the mechanism of HBV-regulated glucose metabolism and IFN production. As shown in Supplementary Fig. 3a, b, HBV increased pyruvate and lactate production. Similar results were obtained when we performed extracellular acidification rate (ECAR) experiments (Supplementary Fig. 3c). Previous studies indicated that hexokinase 2 (HK2) is associated with the voltage-dependent anion channel (VDAC) in the outer membrane of mitochondria, and this association negatively regulates RLR signaling^18,22^. Another previous study showed that protein kinase B (PKB or AKT) mediates hexokinase/VDAC interaction in the membrane of mitochondria^23,24^. Based on our results described above, we hypothesized that HBV might utilize this process. First, we examined the effect of HBV on HK2 expression. Low levels of mitochondrial HK2 were detected in poly(I:C)-transfected cells, whereas the HBV infection restored the HK activity (Supplementary Fig. 3d). Intriguingly, the levels of phosphorylated AKT and HK2 were elevated in mitochondria upon HBV infection (Supplementary Fig. 3e). Because Akt is a serine/threonine kinase^23^, we next examined whether Akt phosphorylated HK2 by using the Akt inhibitor (Akti 1/2). As expected, Akt inhibition resulted in the suppression of the activity and phosphorylation of HK2 (Supplementary Fig. 3f). The effect of VDAC and MAVS on HK activity was further evaluated using shRNAs. Knockdown of VDAC1 or MAVS suppressed the HBV-induced HK activity and mitochondrial HK2 levels (Supplementary Fig. 3g, h). Next, we proceeded to assess the relationship between Akt, VDAC1, HK2, and MAVS during HBV infection. Co-immunoprecipitation (Co-IP) experiments indicated that HBV infection increased Akt associated with HK2 in the early phase, but this association decreased over time (Supplementary Fig. 3i). In contrast, the HBV infection induced the formation of a ternary complex composed of VDAC1, HK2, and MAVS at later times (Supplementary Fig. 3i). Further experiments showed that the Akt activity is necessary for the VDAC1/HK2/MAVS complex formation (Supplementary Fig. 3j). We then interrogated the role of VDAC1/HK2/MAVS complex on glucose metabolism. As shown in Supplementary Fig. 3k, l, Akt inhibition and MAVS knockdown lead to the suppression of ECAR, pyruvate, and lactate production. Previous studies indicated that glucose-6-phosphate (G6P) released HK2 from mitochondria, and the peptide (HKVBD) disrupted VDAC1/HK2 interaction^18,25^. We further examined the role of HK2 mitochondrial location and VDAC1/HK2 interaction in HBV modulated glucose metabolism. As shown in Supplementary Fig. 3m, n, ECAR, pyruvate, and lactate production were reduced in cells treated with G6P and HKVBD.

**Fig. 2:**
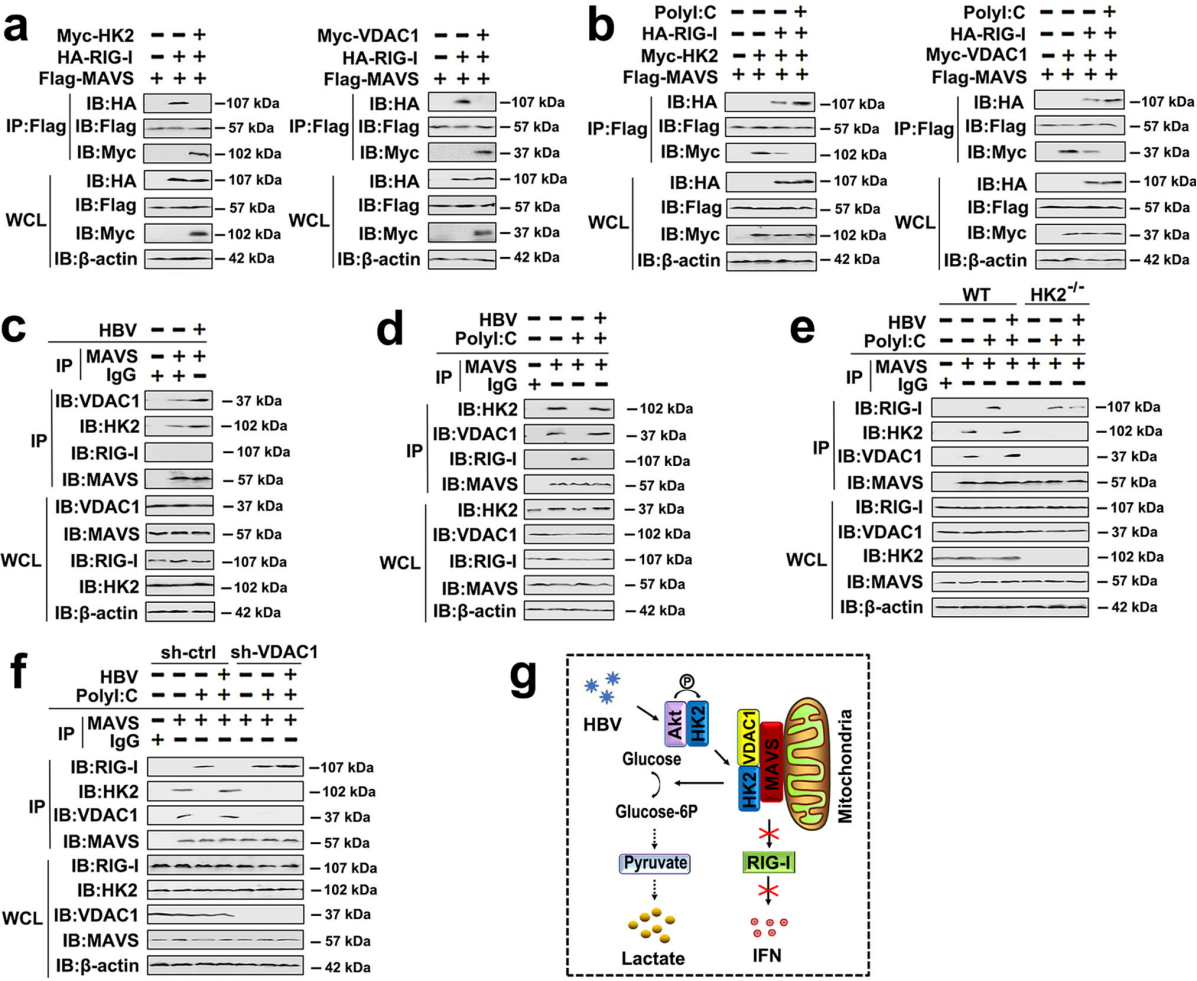
HBV induces the formation of VDAC1/HK2/MAVS sequestering MAVS preventing RIG-I signaling. **a** HepG2 cells were transfected with indicated plasmids for 48 h. Co-IP and immunoblot analyses were performed with the indicated antibodies. **b** HepG2 cells were transfected with indicated plasmids for 36 h and transfected with or without poly(I:C) (1 μg/mL) for 12 h. Co-IP and immunoblot analysis were performed with the indicated antibodies. **c** HepG2-NTCP cells were infected with or without HBV virions (MOI = 300) for 6 days. Co-IP and immunoblot analysis were performed with the indicated antibodies. **d** HepG2-NTCP cells were infected with or without HBV virions (MOI = 300) for 5 days and transfected with or without poly(I:C) (1 μg/mL) for 1 days. Co-IP and immunoblot analysis were performed with the indicated antibodies. **e** HK2 WT (WT) and HK2^−/−^ cells were transfected with control vector or pHBV-1.3 (genotype D) for 36 h and transfected with or without poly(I:C) (1 μg/mL) for 12 h. Co-IP and immunoblot analysis were performed with the indicated antibodies. **f** HepG2-NTCP cells were infected with or without HBV virions (MOI = 300) for 4 days. Then, cells were transfected with indicated plasmids and shRNAs for 36 h and transfected with or without poly(I:C) (1 μg/mL) for 12 h. Co-IP and immunoblot analysis were performed with the indicated antibodies. **g** A hypothetical model for the relationship between HK2, VDAC1, MAVS, and RIG-I in response to poly(I:C) treatment (left panel) or HBV infection (right panel). All experiments were repeated at least three times. See also Supplementary Figs. 3 and 4.

We next explored the mechanisms underlying the HK2 recruitment to mitochondria during HBV infection. Competitive Co-IP experiments demonstrated that both HK2 and VDAC1 disturb the interaction between MAVS and RIG-I by association with MAVS (Fig. 2a). Interestingly, RIG-I could in turn disrupt HK2/MAVS or VDAC1/MAVS interactions, and poly(I:C) plus RIG-I abolished the formation of HK2/MAVS and VDAC1/MAVS interactions (Fig. 2b). We further investigated whether HBV regulates those complexes. Endogenous Co-IP experiments showed that HBV enhances HK2/MAVS as well as VDAC1/MAVS interactions (Fig. 2c). In contrast, poly(I:C) inhibited HK2/MAVS and VDAC1/MAVS interactions. However, the HBV infection restored the HK2/MAVS and VDAC1/MAVS interactions (Fig. 2d). We further showed that HBV impairs the association of MAVS and RIG-I through HK2 and VDAC1 by using HK2^−/−^ cells or transfecting a VDAC1-specific shRNA (Fig. 2e, f). Interestingly, MAVS did not interact with HK2 in VDAC1-shRNA-transfected cells, and MAVS also did not interact with VDAC1 in HK2-deficient cells, suggesting that HK2 and VDAC1 are indispensable for the HK2/VDAC1/MAVS complex formation (Fig. 2e, f).

To further test whether HBV regulates IFN production through mitochondrial HK2/VDAC1 interactions, we first determined the role of this complex on poly(I:C)-induced IFN-β production. As shown in Supplementary Fig. 4a, b, higher IFN-β production was observed in sh-VDAC1-transfected cells and HK2-deficient cells, compared with control cells. Similar results were observed in G6P- and HKVBD-treated cells (Supplementary Fig. 4c, d). The HK2 knockout and the VDAC1 knockdown promoted poly(I:C)-induced IFN-β mRNA expression and enhanced poly(I:C)-induced Mx1 and RSAD2 mRNA expression (Supplementary Fig. 4e, f). Although the inhibitory effect was weakened, HBV still significantly inhibited IFN-β induction (Supplementary Fig. 4e, f), indicating the existence of additional mechanisms used by HBV to suppress RLR signaling. Similar results were observed in G6P- and HKVBD-treated cells (Supplementary Fig. 4g, h). Based on these results, we propose the following model: at the early stage of HBV infection, Akt interacts, and activates HK2. Then, the activated HK2 associated with MAVS and VDAC1 to form a ternary complex in mitochondria. As a result, lactate production is induced, the MAVS-RIG-I interaction is disrupted, and IFN-β production is suppressed (Fig. 2g).

### HBV inhibits RLR signaling exploiting anaerobic glycolysis

**Fig. 3:**
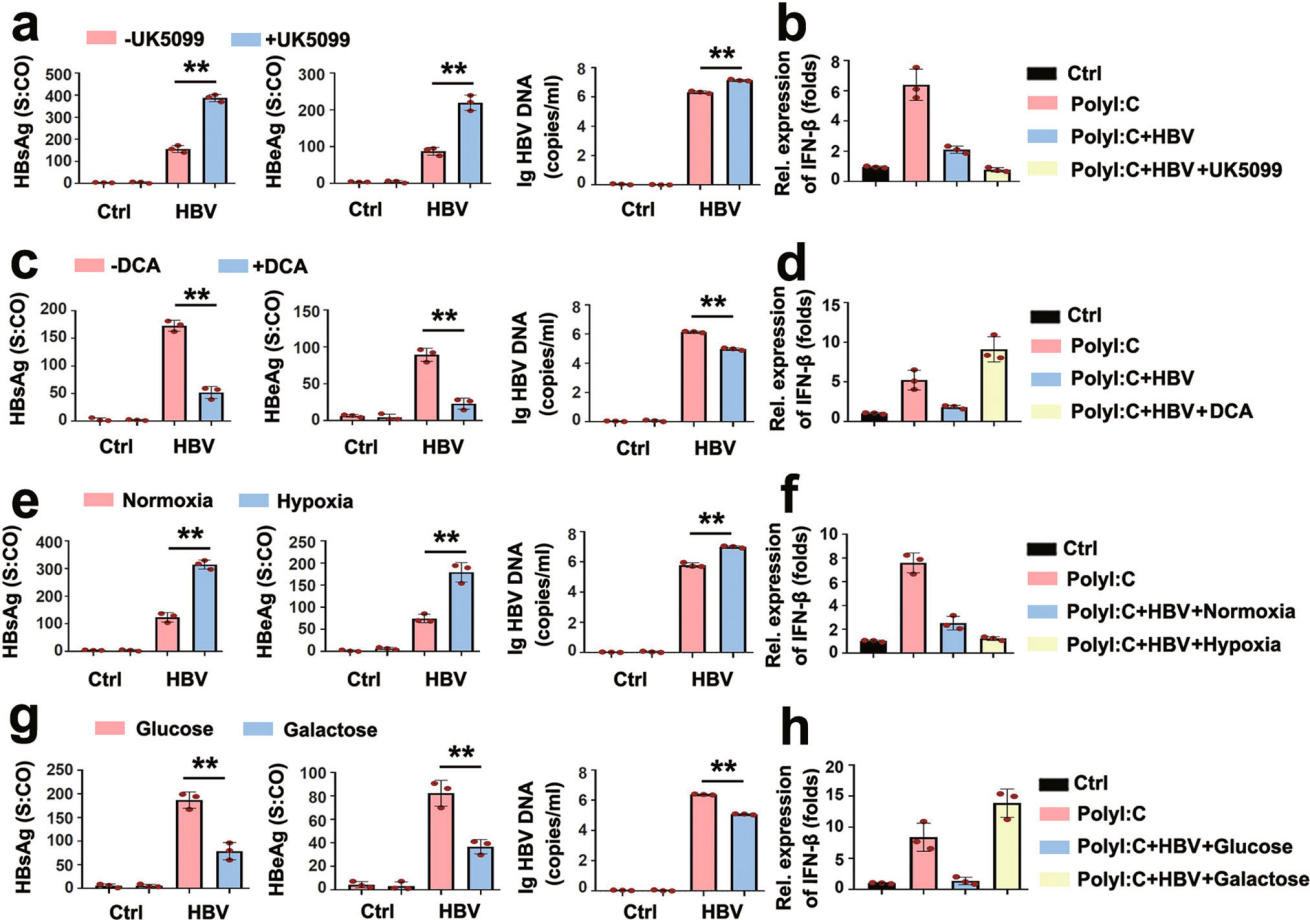
HBV inhibits IFN production by anaerobic glycolysis. **a** PHHs were infected with HBV (MOI = 30) for 8 days and treated with or without UK5099 (10 μM) for 12 h. HBsAg (left panel) and HBeAg (middle panel) secreted in culture supernatants were quantified using CMIA, or the levels of HBV genomes in culture supernatants were determined using qPCR (right panels). **b** PHHs were infected with or without HBV virions (MOI = 30) for 8 days, and transfected with or without poly(I:C) (1 μg/mL), treated with or without UK5099 (10 μM) for 12 h, and subjected to qPCR analyses for IFN-β. **c**, **d** Experiments were performed as in **a** and **b**, except that PHHs were treated with or without DCA (10 mM) for 12 h. **e**, **f** Experiments were performed as in **a**, **b**, except that PHHs were exposed to normoxia (20% O_2_) or hypoxia (1% O_2_) for 12 h. **g**, **h** Experiments were performed as in **a**, **b**, except that PHHs were cultured in mediums containing glucose (25 mM) or galactose (25 mM) for 12 h. All data represent the means ± SD (Student’s *t* test) (**P < 0.01; *P < 0.05, n.s., not significant). *S:CO* signal to cutoff ratio. See also Supplementary Figs. 5.

Oxidative phosphorylation (OxPhos) and anaerobic glycolysis are the two major catabolic glucose pathways^26^. To dissect the key step of glucose metabolism involved in the HBV-mediated regulation of type I IFN production, we employed various methods to regulate the glucose metabolism by preferentially inducing OxPhos or anaerobic glycolysis. Treatment of PHHs and HepG2.2.15 cells with UK5099, which is known as a pyruvate transporter inhibitor, increased HBsAg, HBeAg, and HBV-DNA levels in supernatants (Fig. 3a; Supplementary Fig. 5a, b). HBV and UK5099 synergistically inhibited poly(I:C)-induced IFN-β expression (Fig. 3b). Dichloroacetate (DCA), which controls anaerobic glycolysis shift to OxPhos, decreased HBsAg, HBeAg, and HBV-DNA levels in supernatants (Fig. 3c). Similar results were obtained in DCA-treated HepG2.2.15 cells (Supplementary Fig. 5c, d). As expected based on above-mentioned experiments, these antiviral effects were associated with an attenuation of the HBV-induced inhibition of poly(I:C)-induced IFN-β expression in DCA-treated PHHs (Fig. 3d). Because hypoxia treatment promotes anaerobic glycolysis, whereas galactose treatment enhanced OxPhos^18,27^, we explored the role of hypoxia and galactose on HBV-regulated IFN-β expression. As shown in Fig. 3e, 3f, Hypoxia caused elevated levels of HBsAg, HBeAg, and HBV-DNA in supernatants, accompanied by lower IFN-β expression levels (Fig. 3e, f). Hypoxia also enhanced HBsAg, HBeAg, and HBV-DNA levels in cell culture supernatants and intracellular HBV replication intermediates in HepG2.2.15 cells (Supplementary Fig. 5e, f). In accordance with our other experiments, galactose diminished HBsAg, HBeAg, and HBV-DNA levels in supernatants (Fig. 3g). Consistent with decreased HBV replication, galactose abolished the inhibitory effect of HBV on IFN-β expression upon poly(I:C) transfection (Fig. 3h). We also examined the effects of galactose on HBV in HepG2.2.15 cells and showed that galactose caused lower HBsAg, HBeAg, and HBV-DNA levels in cell culture supernatants and intracellular HBV replication intermediates (Supplementary Fig. 5g, h). Taken together, these results show that HBV manipulates the cell metabolism toward lactate production by glycolysis. The HBV replication benefits from all conditions that stimulate lactate production, whereas conditions that reduce lactate concentrations diminish HBV replication. Simultaneously, lactate levels negatively correlated with the induction of IFN-β. This raised the question concerning cause and effect. We employed IFNAR1-deficient cells to decipher if the proviral effect of lactate is a functional consequence of its inhibitory function on IFN-I induction. As shown in Supplementary Fig. 5i, j, UK5099 increased HBV replication and DCA decreased HBV replication in IFN responsive cells, whereas neither UK5099 nor DCA affected HBV replication in IFNAR1-deficient cells. Similarly, hypoxia-induced HBV replication and galactose reduced HBV replication; however, neither hypoxia nor galactose affected HBV replication in cells lacking IFNAR1 (Supplementary Fig. 5k, l). Taken together, these data indicate that HBV activates anaerobic glycolysis to increase lactate production. Lactate in turn acts proviral by interfering with IFN-β expression.

### Lactate production is crucial for HBV-mediated immune escape

**Fig. 4:**
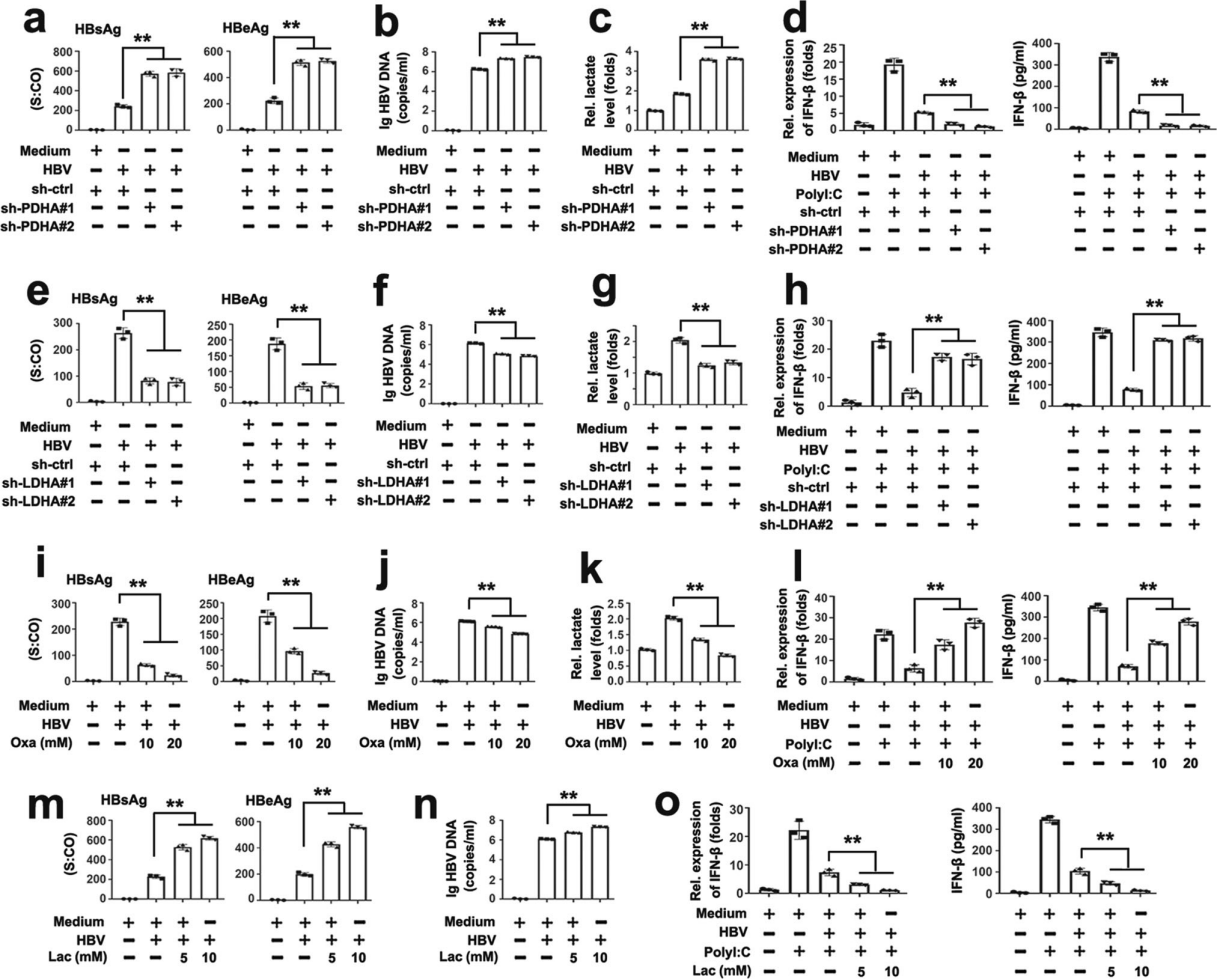
HBV inhibits IFN production by LDHA-dependent lactate production. **a** HepG2-NTCP cells were infected with or without HBV virions (MOI = 300) for 3 days, and transfected the indicated plasmids or shRNAs for 48 h. HBsAg (left panel) and HBeAg (right panel) secreted in culture supernatants were quantified using CMIA. **b**, **c** Experiments were performed as in **a**, except the levels of HBV genomes in culture supernatants were determined using qPCR (**b**), or lactate secretion in supernatants was analyzed (**c**). **d** HepG2-NTCP cells were infected with or without HBV virions (MOI = 300) for 3 days. Cells were transfected with the indicated plasmids or shRNAs for 36 h, and transfected with or without poly(I:C) (1 μg/mL) for 12 h, and subjected to qPCR (left panel) and ELISA (right panel) analyses. **e**–**h** Experiments were performed as in **a**–**d**, except sh-LDHAs were used. **i** HepG2-NTCP cells were infected with or without HBV virions (MOI = 300) for 3 days and treated with or without sodium oxamate for 24 h. HBsAg (left panel) and HBeAg (right panel) secreted in culture supernatants were quantified by CMIA. **j**, **k** Experiments were performed as in **i**, except the levels of HBV genomes in culture supernatants were determined using qPCR (**j**), or the lactate secretion in the supernatant was analyzed (**k**). **l** HepG2-NTCP cells were infected with or without HBV virions (MOI = 300) for 3 days and treated with or without sodium oxamate. Twelve hours later, cells were transfected with or without poly(I:C) (1 μg/mL) for 12 h, and were subjected to qPCR (left panel) and ELISA (right panel) analyses. **m**–**o** Experiments were performed as in **i**, **j**, **l**, except lactate was used. All data represent the means ± SD (Student’s *t* test) (**P < 0.01; *P < 0.05, n.s., not significant). *S:CO* signal to cutoff ratio, *Oxa* oxamate, *Lac* lactate. See also Supplementary Fig. 6.

LDHA and PDHc are two key enzymes that decide the fate of pyruvate. Although LDHA converts pyruvate to lactate, PDHc processes it further, providing acetyl-CoA for the TCA cycle^28^. Pyruvate dehydrogenase A (PDHA), the crucial PDHc subunit, promotes OXPHOS for the TCA cycle^28^. Therefore, we determined whether PDHA or LDHA participate in the HBV-mediated immune escape. We employed two specific shRNAs for PDHA and two specific shRNAs for LDHA and tested their efficiencies (Supplementary Fig. 6a, b). We found a robust increase of HBsAg, HBeAg, and HBV-DNA levels in the supernatants of cells after PDHA knockdown, accompanied by increased lactate levels and a reduction of IFN-β expression upon poly(I:C) challenge (Fig. 4a–d). Conversely, we observed lower levels of HBsAg, HBeAg, and HBV-DNA in cells after the LDHA knockdown compared to control cells, accompanied by a reduction of lactate level and increased IFN-β expression upon poly(I:C) stimulation (Fig. 4e–h). Similar to a LDHA knockdown, the treatment with sodium oxamate, a specific LDHA inhibitor, reduced the HBV replication and HBV-induced lactate levels and abolished the effect of HBV on the IFN-β expression (Fig. 4i–l). We next determined whether lactate is sufficient to affect the HBV replication. Indeed, we observed that lactate supplementation enhanced HBsAg, HBeAg, and HBV-DNA levels in supernatants and reduced the IFN-β expression induced by poly(I:C) (Fig. 4m–o). The effect of sodium oxamate and lactate on HBV replication was not restricted to one cell-type or transformed cells, as similar results were observed in HBV-infected PHHs (Supplementary Fig. 6c, d). Interestingly, an add-back of lactate also rescued the effects of oxamate on HBV replication (Supplementary Fig. 6e). Because cells utilize monocarboxylate transporter 1 (MCT1) for lactate uptake, we knocked down MCT1 in cells to determine whether lactate uptake was crucial for its effect. Lactate induced HBV replication and inhibited IFN-β expression; however, this effect was markedly reduced by an MCT1 knockdown (Supplementary Fig. 6f–h), suggesting that lactate transport into cells is essential for HBV-mediated immune escape. Similarly, oxamate reduced HBV replication and lactate induced HBV replication in WT cells; however, the effect of oxamate and lactate on HBV replication was abolished in IFNAR1-deficient cells (Supplementary Fig. 6I–K), suggesting that oxamate and lactate regulate HBV replication largely through IFN-I. Taken together, these results suggest that lactate serves as a key metabolite mediating the inhibitory effect of HBV on RLR signaling.

### HBV inhibits RLR signaling through lactate in vivo

**Fig. 5:**
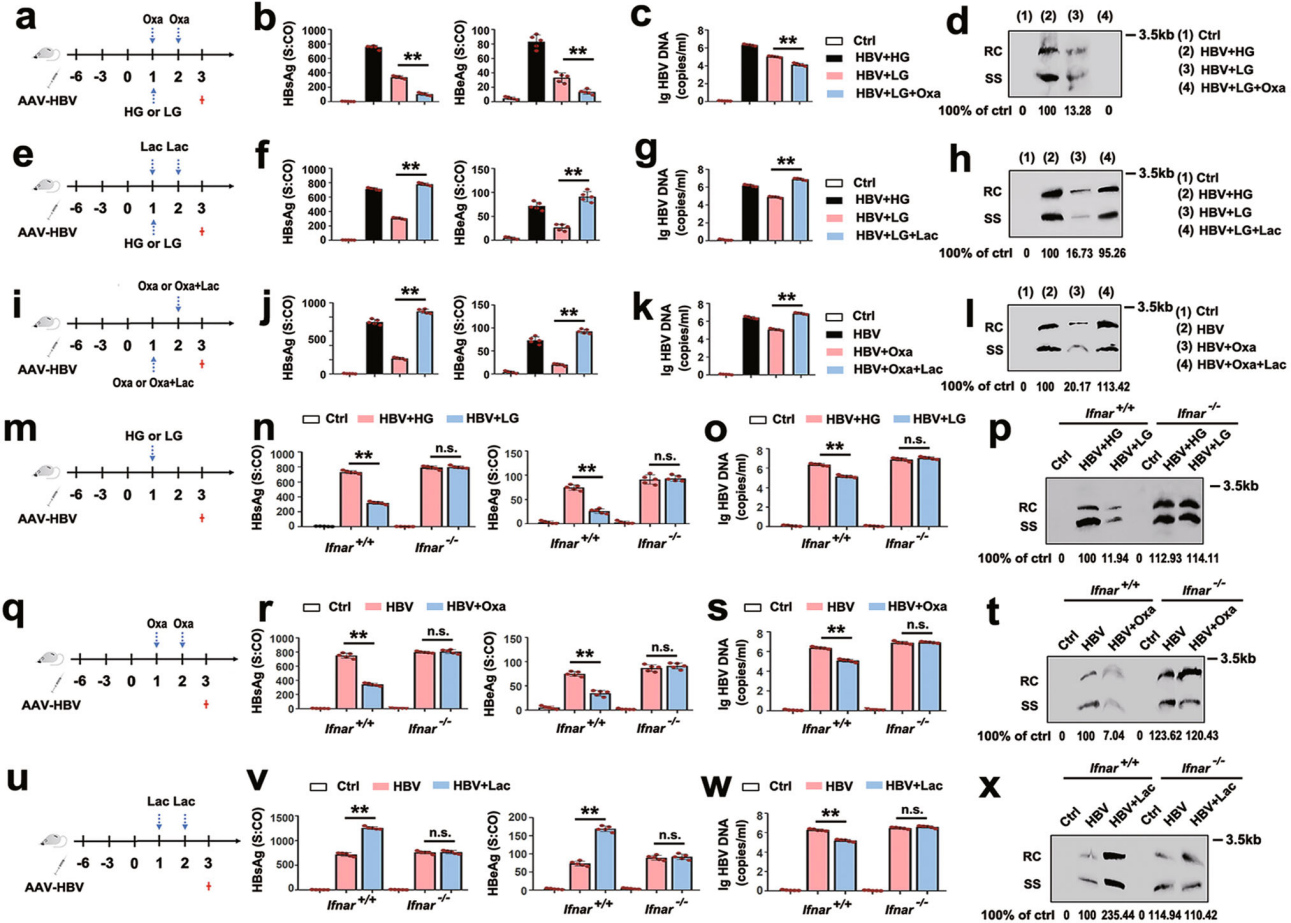
HBV inhibits RLR signaling via LDHA-associated lactate in vivo. **a**–**d** C57BL/6 mice (*n* = 5 for each group) received HI with 10 µg of plasmid pAAV-HBV1.2. At 7 days after HI, the mice were treated with high glucose (1.5 g/kg) or low glucose (0.2 g/kg) with or without injection of sodium oxamate (750 mg/kg) for 2 days (**a**). Serum HBsAg and HBeAg were analyzed using CMIA (**b**). Serum HBV DNA was quantified by qPCR (**c**). HBV replicative intermediates in liver tissues were extracted and detected by Southern blotting (**d**). **e**–**h** Experiments were performed as in **a**–**d**, except sodium lactate (1 g/kg) was used. **i**–**l** Experiments were performed similar as in **a**–**d**, except sodium oxamate (750 mg/kg) and sodium lactate (1 g/kg) were used. **m**–**p** WT (*n* = 5) and IFNAR^−/−^ (*n* = 5) mice received HI with 10 µg of plasmid pAAV-HBV1.2. At 7 days after HI, the mice were treated with high glucose (1.5 g/kg) or low glucose (0.2 g/kg) for 2 days (**m**). Serum HBsAg and HBeAg were analyzed using CMIA (**n**). Serum HBV DNA was quantified by qPCR (**o**). HBV replicative intermediates in liver tissues were extracted and detected by Southern blotting (**p**). **q**–**t** Experiments were performed as in **m**–**p**, except sodium oxamate (750 mg/kg) was used. **u**–**x** Experiments were performed as in **m**–**p**, except sodium lactate (1 g/kg) was used. Data in **b**, **c**, **f**, **g**, **j**, **k**, **n**, **o**, **r**, **s v**, **w** represent means ± SEM (one-way ANOVA) (**P < 0.01; *P < 0.05, n.s., not significant). See also Supplementary Fig. 7.

Having shown that lactate has an important role in HBV-regulated immune escape in vitro, we next studied these mechanisms in the HBV HI mouse model. We observed that HBV infection induces pyruvate and lactate production (Supplementary Fig. 7a, b) in vivo. Remarkably, low glucose treatment and administration of sodium oxamate reduced lactate production and inhibited HBsAg, HBeAg, and HBV-DNA levels in the serum and HBV replication intermediates in mouse liver tissues (Fig. 5a–d; Supplementary Fig. 7c). Simultaneously, the administration of sodium oxamate to mice promoted the induction of IFN-β and IL-6 following a poly(I:C) injection (Supplementary Fig. 7d, e). By contrast, the administration of sodium lactate to mice in addition to low glucose treatment-induced HBsAg, HBeAg, and HBV-DNA levels in the serum and HBV replication intermediates in liver tissues as well as low levels of IFN-β and IL-6 production after poly(I:C) injection (Fig. 5e–h; Supplementary Fig. 7f, g). Administration of sodium oxamate to mice alone inhibited HBV replication and promoted IFN-β and IL-6 production, whereas a lactate add-back compromised the effects of oxamate (Fig. 5i–l; Supplementary Fig. 7h, i).

To test if the lactate produced by LDHA controls HBV replication through IFN-dependent pathways, WT (*Ifnar1*^*+/+*^) and IFNAR1-deficient (*Ifnar1*^*−/−*^) mice were fasted overnight to reduce basal glucose and lactate levels and then treated with high glucose or low glucose. As shown in Fig. 5m–p, low-glucose challenges inhibited HBsAg, HBeAg, and HBV-DNA levels in the serum and HBV replication intermediates in liver tissues of *Ifnar1*^*+/+*^ mice. However, the effect of low glucose on HBV replication was largely abrogated in *Ifnar1*^*−/−*^ mice. Consistently, sodium oxamate decreased HBV replication and sodium lactate increased HBV replication in *Ifnar1*^*+/+*^ mice, whereas the impact of oxamate and lactate on HBV replication was also abolished in *Ifnar1*^*−/−*^ mice (Fig. 5q–x). These results indicate that HBV utilizes lactate produced by LDHA to escape from innate immune recognition and subsequent IFN production in vivo.

### HBV regulates RLR signaling by exposing MAVS to lactate

**Fig. 6:**
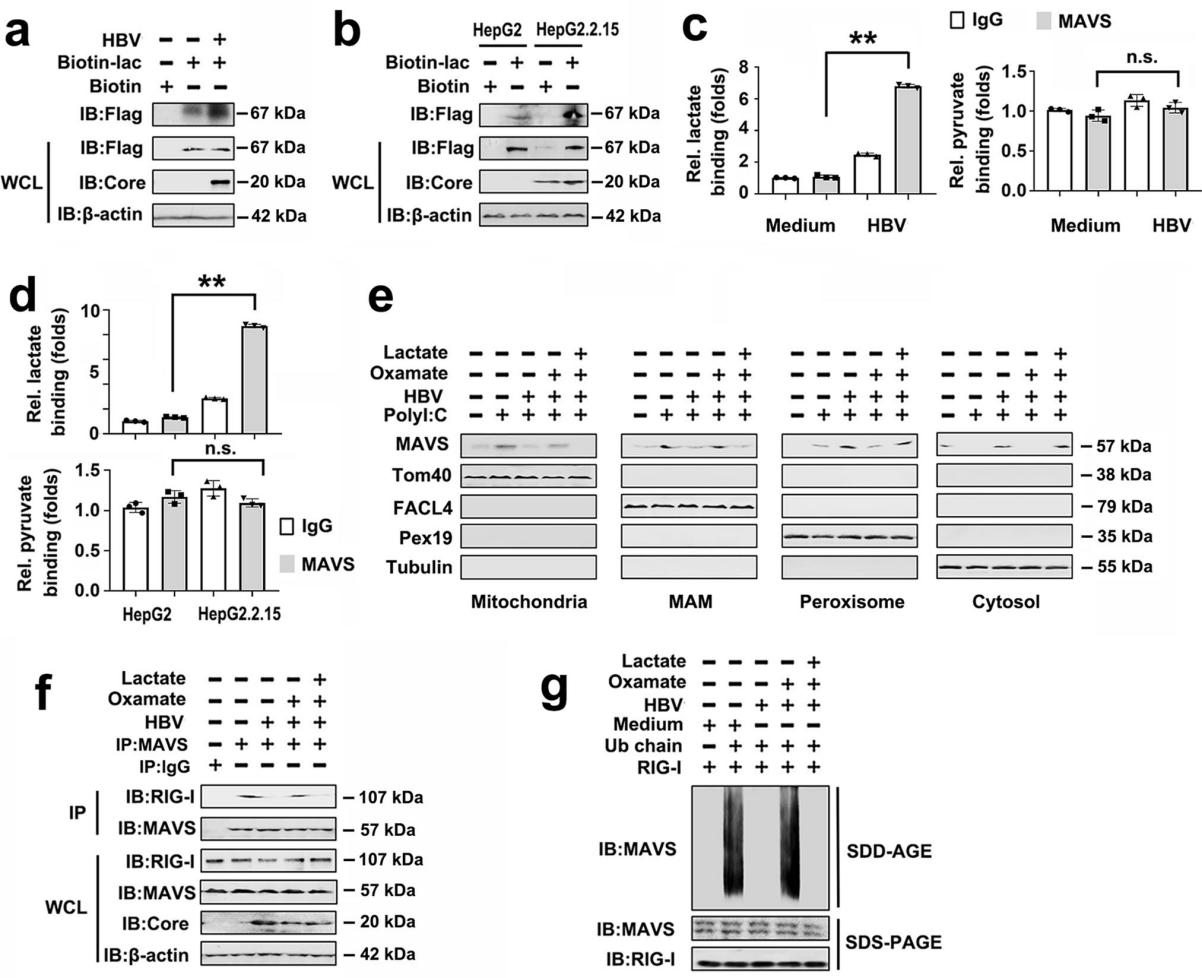
HBV inhibits RLR signaling via promoting lactate/MAVS interaction. **a** HepG2-NTCP cells were infected with or without HBV virions (MOI = 300) for 5 days, and transfected with control vector or Flag-MAVS for 48 h. Immunoblot analysis of binding complexes isolated from cell extracts incubated with biotin-labeled lactate or biotin control. **b** Experiments were performed as in **a**, except HepG2 cells and HepG2.2.15 cells were used. **c** HepG2-NTCP cells were infected with or without HBV virions (MOI = 300) for 7 days. Fluorescence analysis of lactate (left panel) or pyruvate (right panel) binding in complexes IP by IgG or anti-MAVS antibody. **d** Experiments were performed as in **c**, except HepG2 cells and HepG2.2.15 cells were used. **e** HepG2-NTCP cells were infected with or without HBV virions (MOI = 300) for 5 days and transfected with or without poly(I:C) (1 μg/mL) for 12 h. Then, cells were treated with or without sodium oxamate (20 mM) and treated with or without lactate (10 mM) for 24 h. Subcellular fractions were isolated for immunoblot analysis. Fractionation markers: mitochondria (Tom40); MAM (FACL4); peroxisomes (Pex19); cytosol (Tubulin). **f** HepG2-NTCP cells were infected with or without HBV virions (MOI = 300) for 5 days and treated with or without sodium oxamate (20 mM) and then treated with or without lactate (10 mM). Twenty-four hours later, coimmunoprecipitation and immunoblot analysis were performed with the indicated antibodies. **g** GST-RIG-I(N) was incubated with K63-Ub4 and then with mitochondria isolated from HepG2-NTCP cells infected with HBV virions and preincubated with or without oxamate or lactate, followed by the analysis of mitochondria extracts using SDD-AGE (upper panel) and SDS-PAGE (lower panel). Data in **c**, **d** represent means ± SD (Student’s *t* test). See also Supplementary Fig. 8.

We next explored the mechanisms by which lactate regulates RLR signaling in HBV-infected cells. Based on a previous landmark publication that showed that lactate negatively regulated RLR activation by targeting MAVS^18^, we suspected that HBV similarly promotes lactate production to antagonize MAVS by a direct mechanism. Biotin pull-down assay indicated that lactate specifically interacted with MAVS, and this association increased after stimulation with HBV (Fig. 6a). Consistently, the lactate/MAVS interaction was enhanced in HBV-positive HepG2.2.15 cells as compared to HBV-negative HepG2 cells (Fig. 6b). Furthermore, HBV-induced MAVS to interact with lactate, but not pyruvate, in cells under physiological conditions (Fig. 6c). Similarly, MAVS interacted with lactate, but not pyruvate, in HepG2.2.15 cells, as opposed to HepG2 cells (Fig. 6d). Previous studies demonstrated that MAVS is localized to synapse between peroxisomes, mitochondria, and mitochondrial-associated membranes (MAM)^29,30^. This led us to investigate whether HBV modulates the localization of MAVS through lactate. MAVS was present in MAM, peroxisomes, and mitochondria upon a poly(I:C) challenge, whereas the HBV infection forced MAVS to translocate to peroxisomes and the cytosol (Fig. 6e). In addition, the inhibition of lactate production by oxamate abolished the effect of HBV on the localization of MAVS to peroxisomes and the cytosol (Fig. 6e). However, the addition of lactate induced MAVS to localize to peroxisomes and the cytosol (Fig. 6e). The RIG-I-MAVS interaction in mitochondria is a key event during RLR activation^29,30^. We sought to determine whether lactate regulates RIG-I-MAVS interaction during HBV infection. Endogenous Co-IP experiments demonstrated that oxamate abolished the effect of HBV on the RIG-I/MAVS interaction, but lactate addition disrupted the RIG-I/MAVS complex (Fig. 6f). Recognition of MAVS by activated RIG-I is essential for MAVS aggregation and its downstream activity^31^; therefore, we tested whether HBV affects MAVS aggregation by increased lactate levels. As shown in Fig. 6g, HBV inhibited GST-RIG-I(N)-triggered MAVS aggregation, and oxamate promoted MAVS aggregation even in the presence of HBV; however, a lactate add-back impaired MAVS aggregation.

As HBV exploits HK2 to destabilize the MAVS/RIG-I interaction and HK2 acts upstream of lactate, which inactivates MAVS, we wondered if both effects could act independently. As shown in Supplementary Fig. 8a, B, lactate impaired the poly(I:C)-induced IFN-β production and the MAVS/RIG-I interaction despite the absence of HK2. Intriguingly, HK2 overexpression inhibited poly(I:C)-induced IFN-β production and MAVS/RIG-I interaction despite a blockade of lactate production by oxamate (Supplementary Fig. 8c, d). Thus, HK2 can block the RIG-I MAVS pathway by a combination of lactate dependent and lactate independent mechanisms, whereas lactate acts HK2-independently. Together, these results suggest that the HBV infection induces lactate production. Lactate binds to MAVS, leading to disruption of the RIG-I/MAVS interaction and suppressing mitochondrial localization of MAVS and the downstream induction of IFN and pro-inflammatory cytokines.

## Discussion

**Fig. 7:**
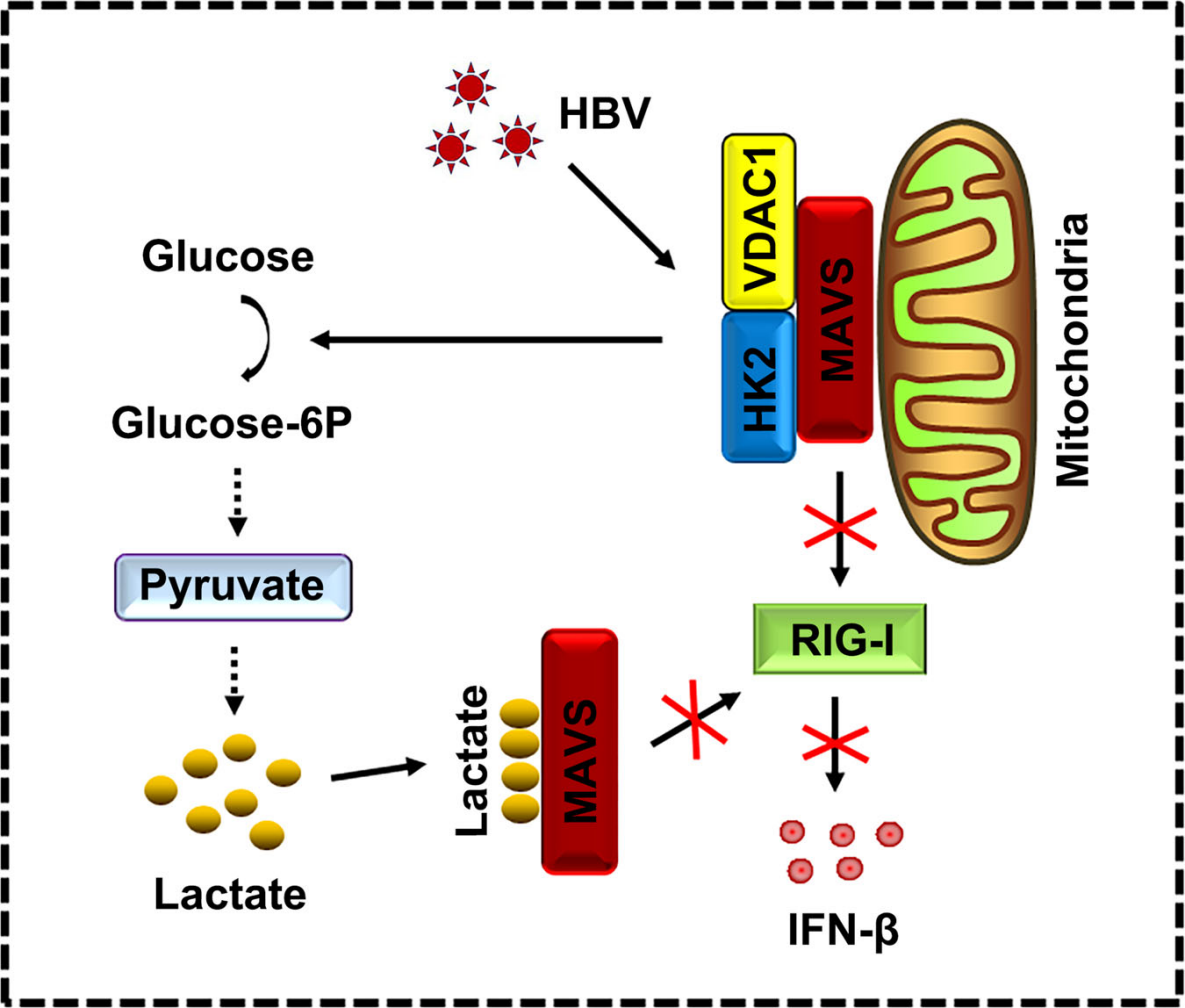
HBV inhibits RLR signaling by HK and lactate. HBV promotes HK activity and lactate production that interacts with MAVS and subsequently inhibits the RIG-I/MAVS interaction and IFN-β production. Solid arrows represent signaling pathways identified in this study. Broken arrows indicate potential signaling pathways. Cross represents signaling pathways inhibited.

We identified a novel mechanism of HBV immune escape, in which HBV increases glucose metabolism and dampens RLR-MAVS signaling (Fig. 7). Mechanistically, HBV promotes HK activity and stimulates LDHA to produce lactate, which in turn interacts with MAVS and subsequently inhibits the RIG-I/MAVS interaction and MAVS aggregation and downstream signaling which would otherwise stimulate IFN production (Fig. 7).

IFN is a critical first line of defense that limits viral gene expression and replication^32^. Energy metabolism is the process of generating energy from nutrients^33^. Recently, studies showed and confirmed an interplay between these two networks during the immune activation in response to viral infections^33–36^. On the one hand, the activated immune system reprograms the metabolic system toward enhanced catabolic activity, leading to a transfer of signals to other systems. On the other hand, elevated catabolic activity is required to meet the increased demand for energy of immune cells. Although some cross-regulation between signaling has been identified, the connection between glucose metabolism and RLR-mediated innate immune activation remained enigmatic. A previous study suggested that glycolysis-derived lactate serves as a threshold to impede IFN production upon RLR activation^18^. The authors further identified MAVS as a molecular hub interconnecting energy metabolism and innate immunity^18^. In the present study, we showed that HBV escapes from the host’s innate immune system by regulating the two connected networks, anaerobic glycolysis, and RLR-MAVS signaling. We further demonstrated that HBV regulates MAVS-RIG-I interaction by a two-pronged attack. (I) HBV promotes a ternary complex composed of HK2, MAVS, and VDAC1 in mitochondria, thereby sequestrating MAVS from RIG-I and preventing the functionally relevant interaction of MAVS with RIG-I. (II) HBV increases lactate levels through LDHA and glycolysis. Lactate physically interacts with MAVS, thereby mislocalizing MAVS from mitochondria and MAM to peroxisomes and the cytosol. Our experiments showed that both inhibitory mechanisms can act independently of each other. We propose the following order of events: initially, HBV induces HK2 translocation to mitochondria for the formation of a ternary complex composed of HK2, MAVS, and VDAC1. Subsequently, HBV stimulates lactate production through anaerobic glycolysis. The lactate binds to MAVS and expels MAVS from mitochondria.

If and how HBV is sensed by the innate immune system has been discussed controversially: some studies indicate that HBV neither stimulates nor not interferes with innate immune responses. This notion has merged in the “stealth virus” concept^37,38^. Other studies demonstrate that RIG-I senses HBV pregenomic RNA to induce interferon production^17^. Several lines of evidence suggest that HBV has developed strategies to suppress host immune responses^39–43^. Our data are clearly in favor of the latter notion that HBV is transiently sensed by the innate immune system but elegantly and efficiently antagonizes its activity.

The function of lactate is intriguing. In the past, lactate was thought to be the useless product of glycolysis. Most cancer cells favor glycolysis for energy metabolism, known as Warburg effect, to enhance their survival and proliferation, accompanied by high lactate generation^44,45^. Several recent studies highlighted the role of lactate not only as a fuel for the TCA cycle in cancer cells, but also as a regulator of the tumor microenvironment and immune cell functions^10,44^. Most oncogenic viruses promote cancer not only via direct insertion of viral DNA into the host genome but also by hijacking the metabolism and cellular physiology. Some viral infections and cancers have similar metabolic demands requiring increased energy metabolism for immune cell function and for proliferation and metastasis of cancer cells^46,47^. Metabolic reprogramming has also been associated with oncogenic viruses such as Epstein–Barr virus, human papillomavirus, and HCV^48^. Our present results suggest that HBV infections—and maybe other viral infections–promote lactate generation in order to limit RLR signaling.

Our data suggest a novel role for lactate during viral infections. Nevertheless, to the best of our knowledge, evading immune surveillance may not be the only function of HBV-induced lactate. It may also support the development of hepatocellular carcinoma. Further studies are needed to address this hypothesis. In summary, our study provides molecular insights into the mechanism of energy metabolism and type I IFN cross-talk during HBV infection, but also suggest an important strategy for the treatment of CHB infection.

## Methods

### Ethics statement

Consistent with the Helsinki Declaration, the collection of clinical samples was approved by the Institutional Review Board of Wuhan University in accordance with guidelines for the protection of human subjects. All study participants provided written informed consent for the collection of samples and subsequent analyses.

All animal experiments were performed in accordance with the National Institutes of Health Guide for the Care and Use of Laboratory Animals. And the protocols and procedures were approved by the Institutional Animal Care and Use Committee of Wuhan University (project license WDSKY0201302).

### Reagents, constructs, and mice

The details of critical reagents are listed in Supplementary Table 1. Reagents unless otherwise specified, all biochemical reagents were purchased from Sigma-Aldrich. The details of antibodies are listed in Supplementary Table 2. The details of shRNAs are listed in Supplementary Table 3. The plasmids pHBV-1.3 (genotype B, Serotype adw, JN406371), Flag-tagged HBs, HBc, HBe, HBp, and HBx (genotype D), and the coding regions of MAVS, HK2 and VDAC1 were created in our laboratory. The plasmid pHBV-1.3 (genotype A, Serotype adw2, HE974381) was kindly provided by Professor Deyin Guo (Wuhan University, China). The plasmid pHBV-1.3 (genotype C, Serotype adr, FJ899793) was kindly provided by Professor Dongping Xu (Beijing 302 Hospital, China). The plasmid IFN-β promoter was kindly provided by Professor Hongbing Shu (Wuhan University, China). The mCherry-GFP-LC3 plasmid was purchased from Addgene (22,418). All the constructs were confirmed by direct DNA sequencing (Sangon Biotech, Shanghai, China), and were then transfected into 293 T cells for expression and analyzed using western blot to verify constructs and the specificity of antibodies. Six- to seven-week-old male wild-type C57BL/6 mice were purchased from the Animal Facility at Wuhan University. Six- to seven-week-old male IFN-αβR^−/−^ C57BL/6 mice were provided by Nanjing Qingzilan Technology Co. Ltd. (Nanjing, China). Mice were housed and handled under specific pathogen-free conditions.

### Cell culture and virus

HepG2.2.15 human hepatoma cells harboring integrated dimers of the HBV genome (GenBank accession number, U95551) were cultured in Dulbecco’s modified Eagle’s medium (DMEM) (Gibco), supplemented with 10% heat-inactivated fetal bovine serum (FBS; Merck Millipore, S0615), 1× MEM non-essential amino acids (NEAA) solution (Gibco, 11,140–035), 100 U/ml penicillin–streptomycin (Gibco, 15,140–122), and 500 µg/ml G418 (Merck Millipore, A2912) at 37 °C in 5% CO_2_. The human hepatoma cell lines Huh7 and HepG2 and human embryonic kidney cells 293 T were obtained from CCTCC (China Center for Type Culture Collection), and grown in DMEM comprising 10% heat-inactivated fetal bovine serum, 100 U/mL penicillin, and 100 µg/mL streptomycin sulfate in 5% CO_2_ humidified at 37 °C.

PHHs were obtained and isolated from freshly resected human liver specimens. Liver cells were prepared under a biosafety hood according to a modified two-step perfusion procedure as previously described. The HBV particles were generated and harvested from HepG2.117 cells, and used for PHH infection. For HBV infection, PHH cells were cultured in primary hepatocyte maintenance medium (PMM) for 24 h and following by infection with HBV particles (MOI = 30) in PMM with 4% PEG 8000 at 37 °C for 24 h. After HBV inoculation, the cells were washed with Phosphate-buffered saline (PBS) for three times and maintained in PMM containing 2% dimethylsulfoxide (Sigma, D5879). The culture medium was renewed every 2 day. HBV replication in PHH was analyzed by chemiluminescence immunoassay (CMIA) and qPCR (Supplementary Fig. 9a).

HepG2-NTCP were maintained in DMEM plus 10% FBS, 1% NEAA 2 mM l-gutamine, 1% penicillin/streptomycin, and 30 μg/ml blasticidin S HCl (Thermo Scientific, Waltham, USA). The HBV particles used for HepG2-NTCP infection were generated and harvested from HepG2.2.15 cells. Cell culture-derived HBV (HBVcc) from the supernatant of HepG2.2.15 was concentrated cells using centrifugal filter devices (Centricon Plus-70, Biomax 100.000, Millipore Corp., Bedford, MA) and titered by HBV-DNA qPCR. HBV replication in HepG2-NTCP was analyzed by CMIA and qPCR (Supplementary Fig. 9b).

### Hydrodynamic injection

In all, 10 μg of pAAV-HBV plasmid were hydrodynamically delivered within 4–6 s into the mouse tail vein in a volume of PBS equivalent to 10% of mouse body weight. HBV replication was analyzed by CMIA and qPCR (Supplementary Fig. 9c).

### Quantitative RT-PCR

Total RNA was isolated using TRIzol reagent (Invitrogen, Carlsbad, CA) according to the manufacturer’s instructions. Quantitative PCR assays were prepared using SYBR RT-PCR kits (Applied Biosystems) in the ABI StepOne real-time PCR system (Applied Biosystems, Waltham, MA). Primers designed to be specific to the conserved regions of either human or murine genes are listed in Supplementary Table 4. The relative expression of each gene was calculated based on the 2^−ΔΔCt^ method, where the results were normalized to the average Ct value of human GAPDH or mouse β-actin.

### Chemiluminescence immunoassay

The levels of secreted HBsAg and HBeAg were determined using the Architect System and HBsAg and HBeAg CMIA kits (Abbott Diagnostics, 06C3622 and 06C3237) from culture supernatants and mouse sera, according to the manufacturer’s instructions.

### Western blot analysis

Western blot analyses were performed as previously described^49^. In brief, cells were harvested by low-speed centrifugation and washed with PBS. Cells were then lysed in cold RIPA Buffer (Cell Signaling Technology, Boston, MA, USA), and protein concentrations were measured using BCA assays (Cell Signaling Technology, Boston, MA, USA). Protein samples (40 μg each) were separated on 12% SDS polyacrylamide gel electrophoresis (PAGE) and then transferred to nitrocellulose membranes (Bio-Rad). The membranes were incubated for 1 h at room temperature with 1× Tris-buffered saline with Tween 20 and 5% (w/v) non-fat milk to block non-specific binding. Following the membrane incubation at 4 °C overnight with target-specific primary antibodies, blots were incubated with horseradish peroxidase-linked secondary antibodies (Jackson ImmunoResearch) for an additional 1 h and the result was normalized to reference internal control. The signal of immunoreactive bands was visualized using a LAS-4000 image document instrument (FujiFilm, Tokyo, Japan).

### Measurement of hexokinase activity and lactate levels

Mitochondria were isolated by using the Mitochondria Isolation kit (Thermo 89874), and pellets were lysed and subjected to hexokinase activity measurement using a Hexokinase Colorimetric Assay kit (Biovision K789-100). Lactate Plus Test Strips (Nova Biomedical/Fisher) were used to measure secreted lactate levels. And a lactate Colorimetric/ Fluorometric Assay kit (Biovision K607-100) were used for intracellular lactate levels measurement according to the manufacturer’s protocol. For seahorse analysis, cells with control or MAVS knockdown were prepared, and an XF24 Extracellular Flux analyzer (Seahorse Biosciences, Billerica, MA) was used to measure the ECAR.

### Transfection and luciferase reporter gene assays

Cells plated on 24-well dishes were transfected using Lipofectamine 3000 (Invitrogen) for 24 h, after which they were serum-starved for an additional 24 h prior to harvest. A renilla luciferase reporter vector pRL-TK was used as the internal control of transfection efficiency. Cells were subjected to luciferase activity assays using a dual-specific luciferase assay kit (Promega, Madison, WI, USA). Firefly luciferase activities were normalized on the basis of renilla luciferase activities.

### Southern blot analysis

Cells were lysed at 4 °C with lysis buffer (50 mM Tris-HCl, pH 7.4, 1 mM EDTA, and 1% NP-40) for 10 min. Cell debris and nuclei were removed by centrifugation, and the supernatants were mixed and incubated with 10 mM MgCl_2_ and 100 μg/ml DNase I (Roche, 000000010,104,159,001) at 37 °C for 30 min, and were stopped by the addition of 25 mM EDTA. By adding 0.5 mg/ml proteinase K (Qiagen, 19,133) and 1% SDS, the mixture was incubated at 55 °C for 2 h. Cellular DNA was extracted with a phenol/chloroform (1:1), precipitated with isopropanol, washed with 75% ethanol and resuspended in TE buffer (10 mM Tris-HCl, pH 8.0, 1 mM EDTA). DNA samples were resolved on 1% agarose gels and transferred to positively-charged nylon membranes (GE Healthcare, RPN303B). The membranes were then hybridized with a ^32^P-labeled full-length HBV probes prepared using a random priming labeling kit (GE Healthcare, RPN1633) in hybridization buffer (G-Biosciences, 786–160). Hybridization signals were visualized and analyzed using a Phospho-Imager (Cyclon, Packard Instrument).

### Dye quenched-bovine serum albumin degradation assay

In brief, treated cells were incubated with 10 μg/ml DQ Red bovine serum albumin (BSA) (Invitrogen, D-12,051) for 30 min in humidified at 37 °C. And the fluorescent signal generated by lysosomal proteolysis of DQ Red BSA was quantified with an LSM 710 confocal microscope (Zeiss, Germany) as described above.

### AO staining

In brief, treated cells were stained with 5 μM AO (Sigma, A9231) for 15 min in humidified at 37 °C. And the signal read at 488 nm (green) or 561 nm (red) was detected by confocal microscopy.

### Generation of KO cell lines

The lentiCRISPRv2 plasmid was kindly provided by Jianguo Wu (Wuhan University). A specific oligo targeting the gene was designed using Cas9 target design tools (http://www.genome-engineering.org). The target guide sequence cloning protocol can be found at the Zhang Laboratory GeCKO Web site (http://www.genome-engineering.org/gecko/). HEK-293T cells were co-transfected with the specific lentiCRISPRv2 plasmid, lentivirus packaging plasmid psPAX2, together with envelope plasmid pMD2.G using Lipofectamine 3000. And the lentiviral particles harvested in medium was centrifuged at 15,000 × *g* for 5 min and then filtered through a 0.22-mm filter (Millipore) to remove cells. When recipient cells were grown to ∼70% confluence, they were incubated in fresh culture medium containing 8 mg/ml polybrene. Subsequently, the specific lentiCRISPRv2 lentivirus -containing media was added to the cells. Cells were plated in a 96-wells plate at ~1 cell per well to get a single clone. The monoclonal cell colonies were singled out for enlarged culture. KO cell lines were obtained from these enlarged monoclonal cells, and KO was confirmed by qRT-PCR and western blotting.

### MAVS aggregation assays

For in vitro MAVS aggregation, crude mitochondria were isolated and RIG-I activation was detected as previously described^18^. In brief, 100 ng GST-RIG-I(N) and 50–100 ng ubiquitin chains (K63-Ub4 from Boston Biochem UC-310B) were mixed and incubated in 1 mL buffer containing 20 mM HEPES-KOH (pH 7.0) and 10% (v/v) glycerol at RT for 10 min. And then this reaction mixture was mixed with 10 mg of mitochondrial fraction in 10 mL Buffer B (20 mM HEPES-KOH [pH 7.0], 5 mM MgCl_2_, and 0.25 M d-mannitol) at 30 °C for 30 min. The mitochondria fraction was then pelleted at 10,000 × *g* for 10 min and washed twice with Buffer C (20 mM HEPES-KOH at pH 7.4, 0.5 mM EGTA, 0.25 MD-mannitol, and EDTA-free protease inhibitor cocktail) and subjected to semi-denaturing detergent agarose gel electrophoresis.

### Coimmunoprecipitation and pull-down assays

Coimmunoprecipitation analyses were performed as previously described^50^. In briefly, collected treated cells were lysed using lysis buffer (20 mM Tris, pH 7, 0.5% (vol/vol) Nonidet-P40, 25 mM NaCl, 3 mM EDTA, 3 mM EGTA, 2 mM dithiothreitol, 0.5 mM phenylmethyl sulfonyl fluoride, 20 mM β-glycerol phosphate, 1 mM sodium vanadate and 1 mg/ml of leupeptin). Lysates were mixed and incubated with antibodies or IgG and protein G–agarose beads overnight at 4 °C. Beads were then washed with lysis buffer for 3–5 times, and bound proteins were separated with subsequent immunoblotting analysis using sodium dodecyl sulfate polyacrylamide gel electrophoresis (SDS-PAGE). For biotin-lactate pull-down assays, magic Dynabeads MyOne Streptavidin T1 was preincubated with free biotin or biotin-labeled lactate in PBS for 1 h at RT, and then mixed and incubated with cell lysates overnight on a rotating platform at 4 °C. The beads were washed 3–4 times before analyzed by immunoblotting.

### Immunocytochemical staining

The target cells were incubated and fixed with equal volumes of methyl alcohol and acetone for 15 min, washed with PBS for three times, and blocked with 4% BSA in PBS for 1 h at room temperature. Then, the cells were stained with the primary antibody at 4 °C overnight and subsequently incubated with secondary antibodies (ProteinTech Group, Wuhan, China) for 1 h. By using confocal laser microscopy (FLUOVIEW FV1000; Olympus, Tokyo, Japan), the staining cells were clearly visualized.

### Subcellular fractionation

MAM, mitochondria, and peroxisome were isolated from cells using Percoll density gradient fractionation as described^29^. Equivalent amounts of protein from each fraction were separated by SDS-PAGE and analyzed by immunoblotting.

### Statistical analysis

Data were obtained from three independent reproducible experiments. Data were expressed as mean ± standard deviations or mean ± the standard error of the mean. Statistical significance was determined using Student’s unpaired two-tailed *t* test, or one-way ANOVA multiple comparison test as indicated in the legend. A *p* value < 0.05 was considered significant and was indicated with an asterisk (*).

### Reporting summary

Further information on research design is available in the Nature Research Reporting Summary linked to this article.

## Supplementary information

Supplementary Information

Reporting Summary

## Data Availability

The data supporting the findings of this study are available within the article and its Supplementary Information files or from the corresponding author on reasonable request. Source data are provided with this paper.

## Source data

Source Data
