# Supplementary material for: Hepatitis B virus rigs the cellular metabolome to avoid innate immune recognition
Source: Nat Commun. 2021 Jan 4;12:98. doi: 10.1038/s41467-020-20316-8 (PMC7782485; doi:10.1038/s41467-020-20316-8)
Supplement: Supplementary file 1 — Supplementary Information [file 41467_2020_20316_MOESM1_ESM.pdf]

Supplementary Information

Hepatitis B Virus rigs the cellular metabolome to avoid innate immune recognition

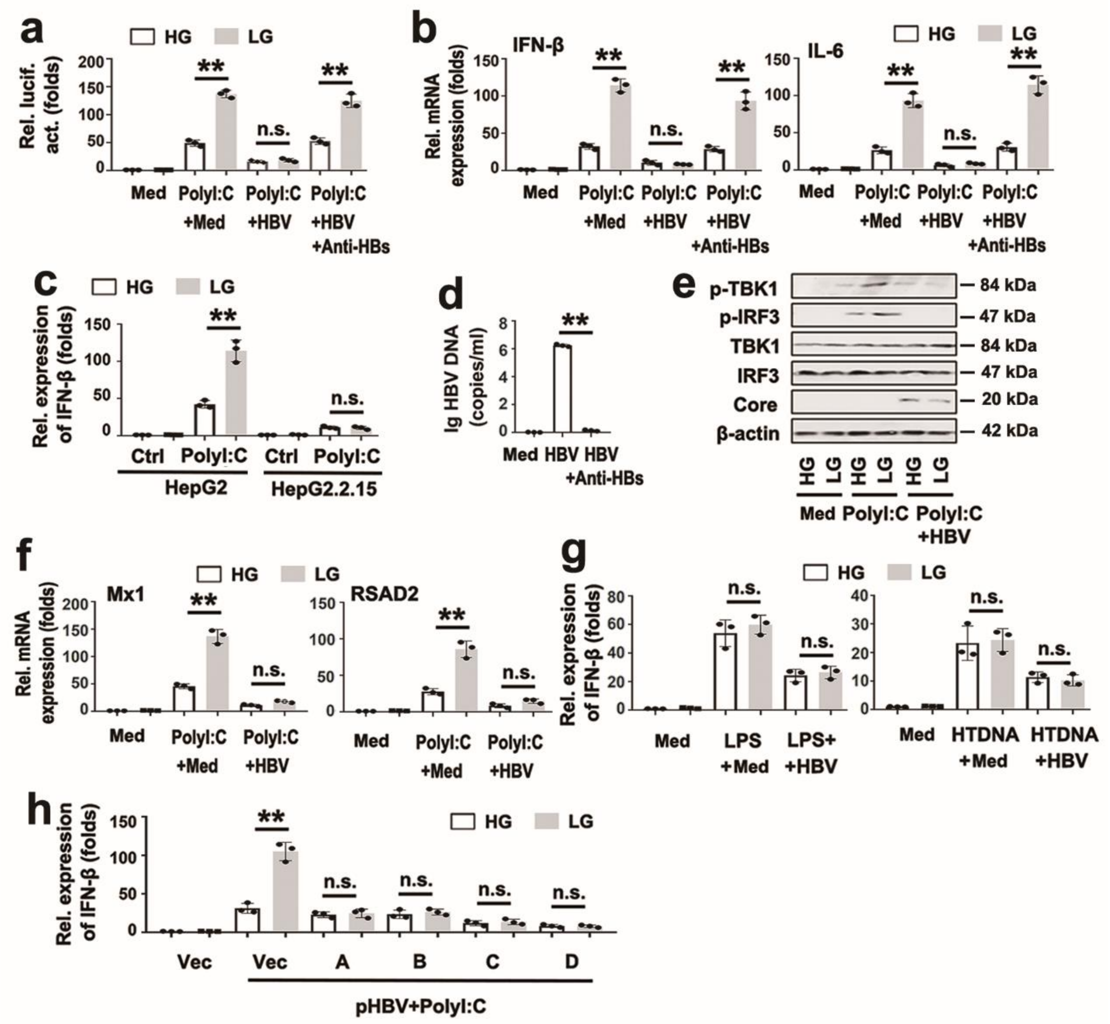

Supplementary Figure 1. HBV inhibits RLR signaling via promoting glucose metabolism, Related to Figure 1.

(a) HepG2-NTCP cells were infected with or without HBV virions (MOI=300) for 5 days. The supernatants were pretreated with or without anti-HBs, an HBV neutralizing antibody. Then, cells were cultured with high glucose (25 mM) or low glucose (5 mM) and transfected with IFN-β promoter and the indicated plasmids for 24 h, transfected with or without poly(I:C) (1 μg/mL) for 12 h, and subjected to luciferase assays.

(b) Experiments were performed as in (A), except IFN-β mRNA (left panel) and IL-6 mRNA (right panel) levels were measured using qPCR.

(c) HepG2 and HepG2.2.15 cells were cultured with high glucose (25 mM) or low glucose (5 mM) for 24 h, transfected with or without poly(I:C) (1 μg/mL) for 12 h, and subjected to qPCR assays.
(d) HepG2-NTCP cells were infected with or without HBV virions (MOI=300) for 5 days. The supernatants were pretreated with or without anti-HBs for 24 h. HBV genomes in culture supernatants were determined by qPCR.
(e) HepG2-NTCP cells were infected with or without HBV virions (MOI=300) for 5 days. Cells were cultured with high glucose (25 mM) or low glucose (5 mM) and transfected with or without poly(I:C) (1 μg/mL) for 12 h, and subjected to western blot assays.
(f) Experiments were performed as in (e), except Mx1 mRNA (left panel) and RSAD2 mRNA (right panel) levels were measured using qPCR.
(g) Experiments were performed as in (b), except LPS (50 ng/ml) (left panel) or HTDNA (1 mg/mL) (right panel) were used.
(h) Huh7 cells were cultured with high glucose (25 mM) or low glucose (5 mM) and transfected with the indicated pHBV-1.3 (genotype A-D) or empty vector for 24 h, transfected with or without poly(I:C) (1 μg/mL) for 12 h, and subjected to qPCR assays.
Data (a-d and f-h) represent means ± SD (Student's t-test) (**P < 0.01; *P < 0.05, n.s., not significant) S:CO, signal to cutoff ratio.

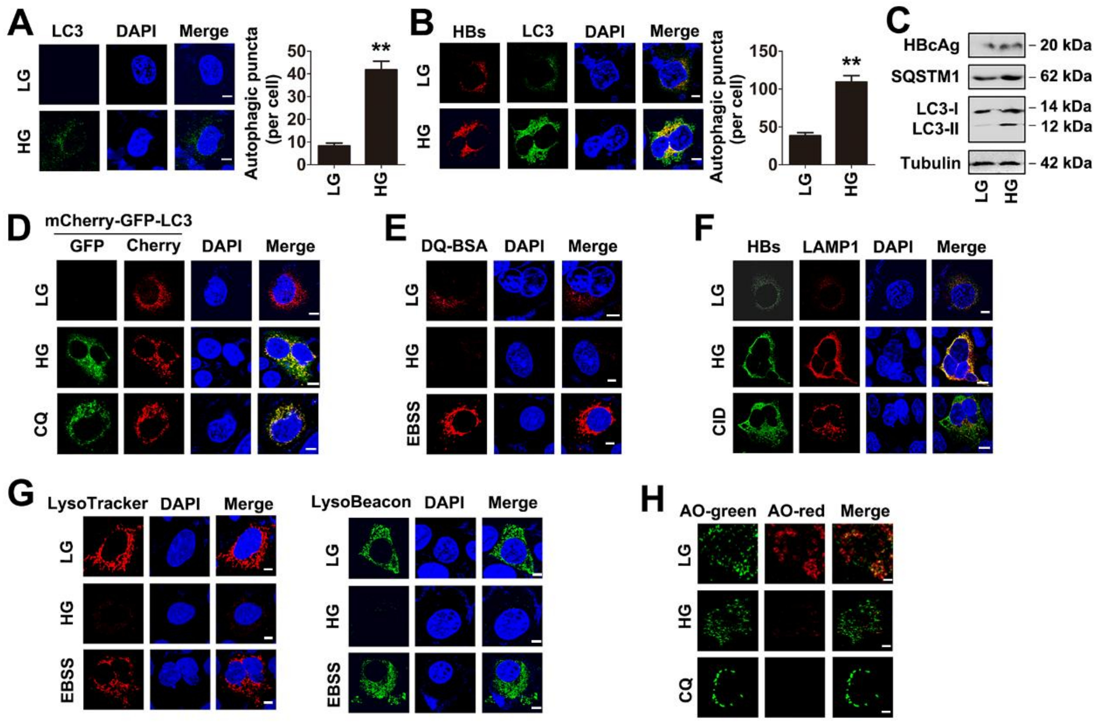

Supplementary Figure 2. Glucose promotes HBV replication by suppressing autophagic degradation. Related to Figure 1.
(a) HepG2 cells were cultured with high glucose (25 mM) or low glucose (5 mM) for 48 h prior to immunofluorescence assays.

(b) HepG2 cells were transfected with control vector or pHBV-1.3 (genotype D) and cultured with high glucose (25 mM) or low glucose (5 mM) for 48 h prior to immunofluorescence assays.
(c) Experiments were performed as in (B), except Western blot analyses were performed.
(d) HepG2 cells were transfected with mCherry-GFP-LC3 plasmid and cultured with high glucose (25 mM) or low glucose (5 mM) for 48 h. The expression of mCherry and GFP was determined by confocal microscopy. Cells cultured with chloroquine (CQ) (10 μM) for 24 h were used as a positive control.
(e) HepG2 cells were treated as in (b), followed by incubation with DQ-BSA (10 μg/ml) for 30 min. The accumulating fluorescent signal of DQ-BSA was analyzed by confocal microscopy. Cells treated with EBSS for 2 h were used as a positive control.
(f) Experiments were performed as in (b). Cells treated with CID1067700 (CID) (5 μM) for 24 h were used as a positive control.
(g) Experiments were performed as in (e), except cells were stained with LysoTracker Red (100 nM) or LysoBeacon Green (1 μM) for 1 h.
(h) Experiments were performed as in (e), except cells were stained with acridine orange (AO) for 15 min. AO fluorescence was detected by confocal microscopy using a 488-nm (green) or a 561-nm (red) laser.
All error bars indicate means ± SD (Student's t-test) (** $P < 0.01$; * $P < 0.05$). Scale bar: 5 μm.

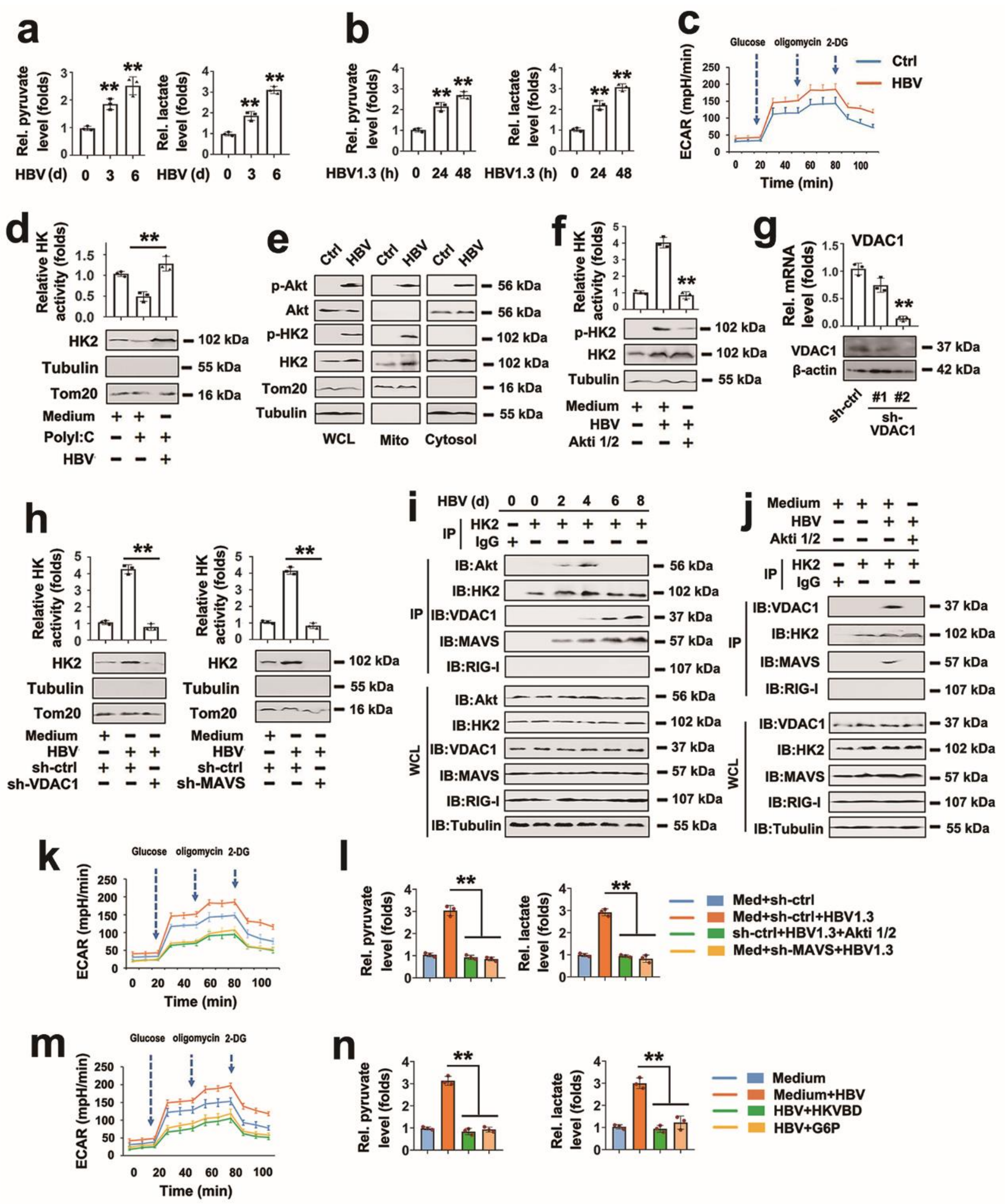

Supplementary Figure 3. HBV induces glucose metabolism through inducing VDAC1/HK2/MAVS interaction, Related to Figure 2.

(a) PHHs were infected with HBV virions (MOI = 30) for the indicated times before measuring total pyruvate level (left panels) and lactate level (right panels) in supernatants.

(b) Huh7 cells were transfected with pHBV-1.3 (genotype D) for the indicated times before measuring total pyruvate levels (left panel) and lactate levels (right panel) in supernatants.

(c) PHHs were infected with HBV virions (MOI = 30) for the indicated times prior to ECAR analyses in supernatants.

(d) HepG2-NTCP cells were infected with or without HBV virions (MOI=300) for 5 days, and transfected with or without poly(I:C) (1 μg/mL) for 12 h, followed by

analysis of mitochondria HK activity (upper panel) and mitochondria HK2 protein levels (lower panel). Fractionation markers: mitochondria (Tom20); cytosol (Tubulin).
(e) HepG2-NTCP cells were infected with or without HBV virions (MOI=300) for 4 days. Subcellular fractions were isolated for immunoblot analysis.
(f) HepG2-NTCP cells were infected with or without HBV virions (MOI=300) for 4 days, and treated with or without Akti 1/2 (1 μM) (AKT1/2 kinase inhibitor) for 2 days, followed by analysis of mitochondria HK activity (upper panel) and whole-cell p-HK2 and HK2 protein levels (lower panel).
(g) HepG2 cells were transfected with shRNA-ctrl or indicated shRNA-VDAC for 48 h prior to qPCR (upper panel) and western blot (lower panel) assays.
(h) Experiments were performed as in (d), except cells were transfected with indicated shRNAs for 24 h.
(i) HepG2-NTCP cells were infected with or without HBV virions (MOI=300) for indicated times. Co-IP and immunoblot analysis were performed with the indicated antibodies.
(j) HepG2-NTCP cells were infected with or without HBV virions (MOI=300) for 4 days, and treated with or without Akti 1/2 (1 μM) for 2 days. Co-IP and immunoblot analysis were performed with the indicated antibodies.
(k) HepG2-NTCP cells were infected with or without HBV virions (MOI=300) for 4 days, and transfected with indicated sh-RNAs or treated with Akti 1/2 (1 μM) for 2 days prior to ECAR analyses.
(l) Experiments were performed as in (k), except total pyruvate level (left panels) and lactate level (right panels) in supernatants were analyzed.
(m and n) Experiments were performed as in (k) and (l), except cells were treated with hexokinase dissociation peptide (HKVBD) (25 μM) or glucose 6-phosphote (G6P) (2 mM) for 1 h.
Data (a, b, d, f, g, h, i and n) represent means ± SD (Student's t-test) (**P < 0.01; *P < 0.05, n.s., not significant)

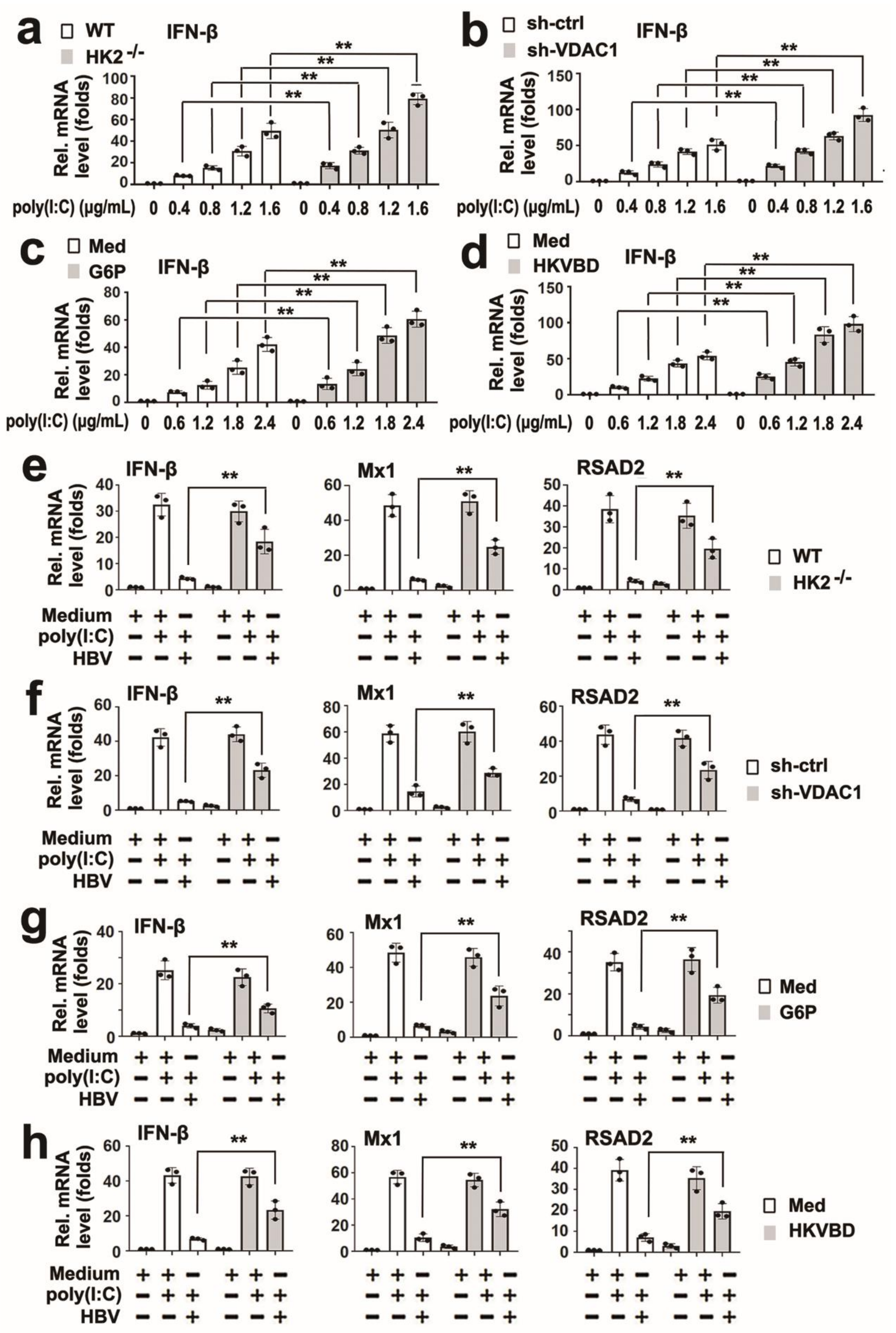

Supplementary Figure 4. HBV inhibits IFN production through inducing VDAC1/HK2/MAVS interaction, Related to Figure 2.

(a) WT or HK2$^{-/-}$ cells were transfected with indicated concentration of poly(I:C) for 12 h prior to qPCR assays.

(b) HepG2 cells were transfected with indicated shRNAs for 36 h and transfected with indicated concentration of poly(I:C) for 12 h prior to qPCR assays.
(c and d) HepG2 cells were transfected with indicated concentration of poly(I:C) for 12 h, and treated with glucose 6-phosphote (G6P) (2 mM) (c) or hexokinase dissociation peptide (HKVBD) (25 μM) (d) for 1 h prior to qPCR assays..
(e) WT or $HK2^{-/-}$ cells were transfected with control vector or pHBV-1.3 (genotype D) for 36 h and transfected with or without poly(I:C) (WT cells 1.2 μg/mL, $HK2^{-/-}$ cells 0.8 μg/mL) for 12 h prior to qPCR assays.
(f) HepG2 cells were transfected with control vector or pHBV-1.3 (genotype D) and indicated shRNAs for 36 h. Then, cells were transfected with or without poly(I:C) (shRNA control transfected cells 1.2 μg/mL, shRNA-VDAC1 transfected cells 0.8 μg/mL) for 12 h prior to qPCR assays.
(g) HepG2 cells were transfected with control vector or pHBV-1.3 (genotype D) for 36 h and transfected with or without poly(I:C) (control cells 1.8 μg/mL, G6P treated cells 1.2 μg/mL) for 12 h. Then, cells were treated with G6P (2 mM) for 1 h prior to qPCR assays.
(h) Experiments were performed as in (g), except cells were treated with HKVBD.
All error bars indicate means ± SD (Student's t-test) (**P < 0.01; *P < 0.05, n.s., not significant)

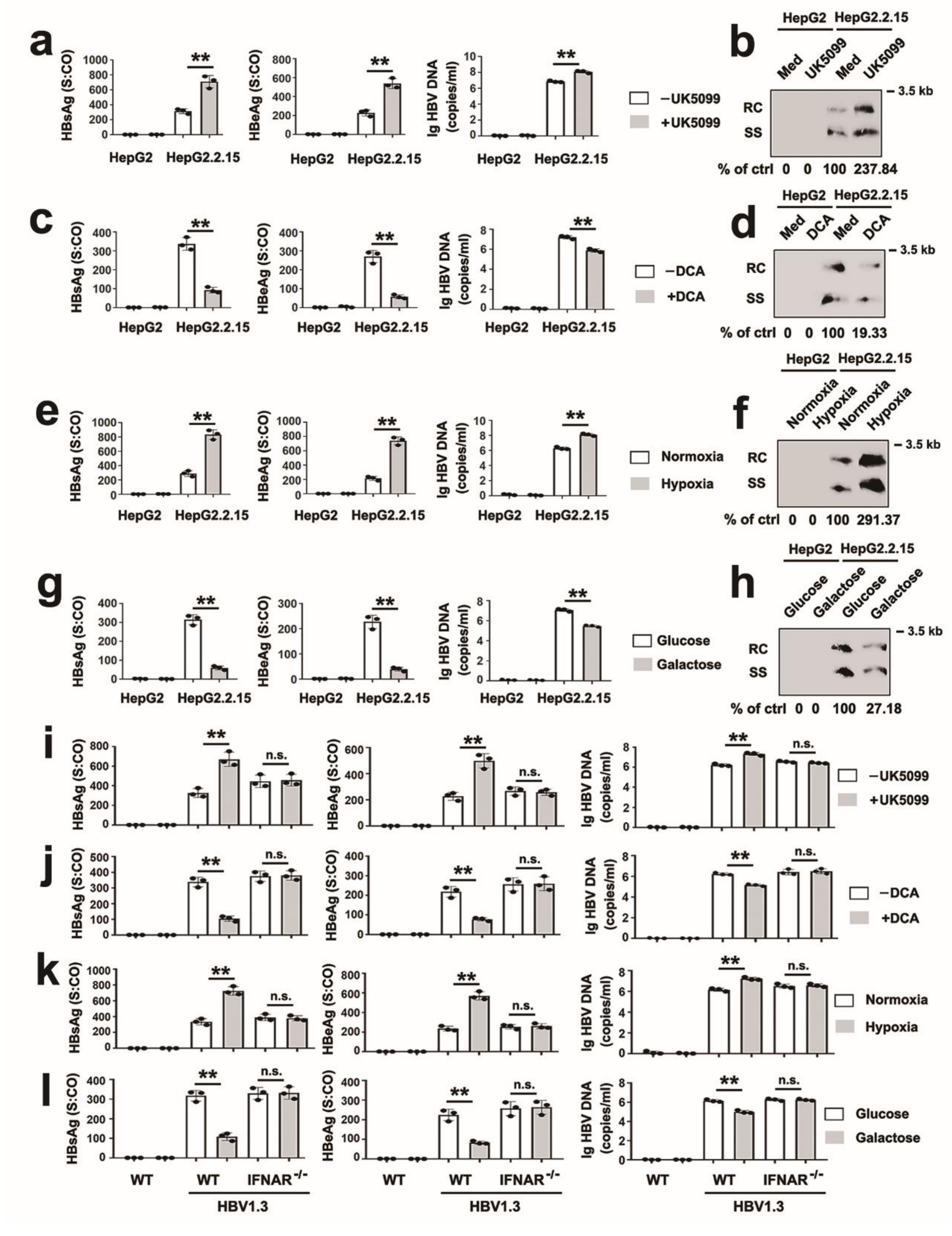

Supplementary Figure 5. HBV inhibits IFN production through anaerobic glycolysis, Related to Figure 3.

(a) HepG2 and HepG2.2.15 cells were treated with or without UK5099 (10 μM) for 12 h. HBsAg (left panel) and HBeAg (middle panel) secreted in culture supernatants were quantified by CMIA, or the levels of HBV genomes in culture supernatants were determined using qPCR (right panels).

(b) Experiments were performed as in (a), except Southern blot analyses were performed.

(c and d) Experiments were performed as in (a and b), except DCA (10 mM) were

used.
(e and f) Experiments were performed as in (a and b), except HepG2 and HepG2.2.15 cells were exposed to normoxia (20% O_2) or hypoxia (1% O_2) for 12 h.
(g and h) Experiments were performed as in (a and b), except HepG2 and HepG2.2.15 cells were cultured in mediums containing glucose (25 mM) or galactose (25 mM) for 12 h.
(i) IFNAR WT (WT) and $IFNAR^{-/-}$ cells were transfected with control vector or pHBV-1.3 (genotype D) for 36 h, treated with or without UK5099 (10 μM) for 12 h. HBsAg (left panel) and HBeAg (middle panel) secreted in culture supernatants were quantified by CMIA, or the levels of HBV genomes in culture supernatants were determined using qPCR (right panels).
(j) Experiments were performed as in (i), except DCA (10 mM) were used.
(k) Experiments were performed as in (i), except WT and $IFNAR^{-/-}$ cells were exposed to normoxia (20% O_2) or hypoxia (1% O_2) for 12 h.
(l) Experiments were performed as in (i), except WT and $IFNAR^{-/-}$ cells were cultured in mediums containing glucose (25 mM) or galactose (25 mM) for 12 h.
Data (a, c, e, g, and i-l) represent means ± SD (Student's t-test) (**$P < 0.01$; *$P < 0.05$, n.s., not significant)

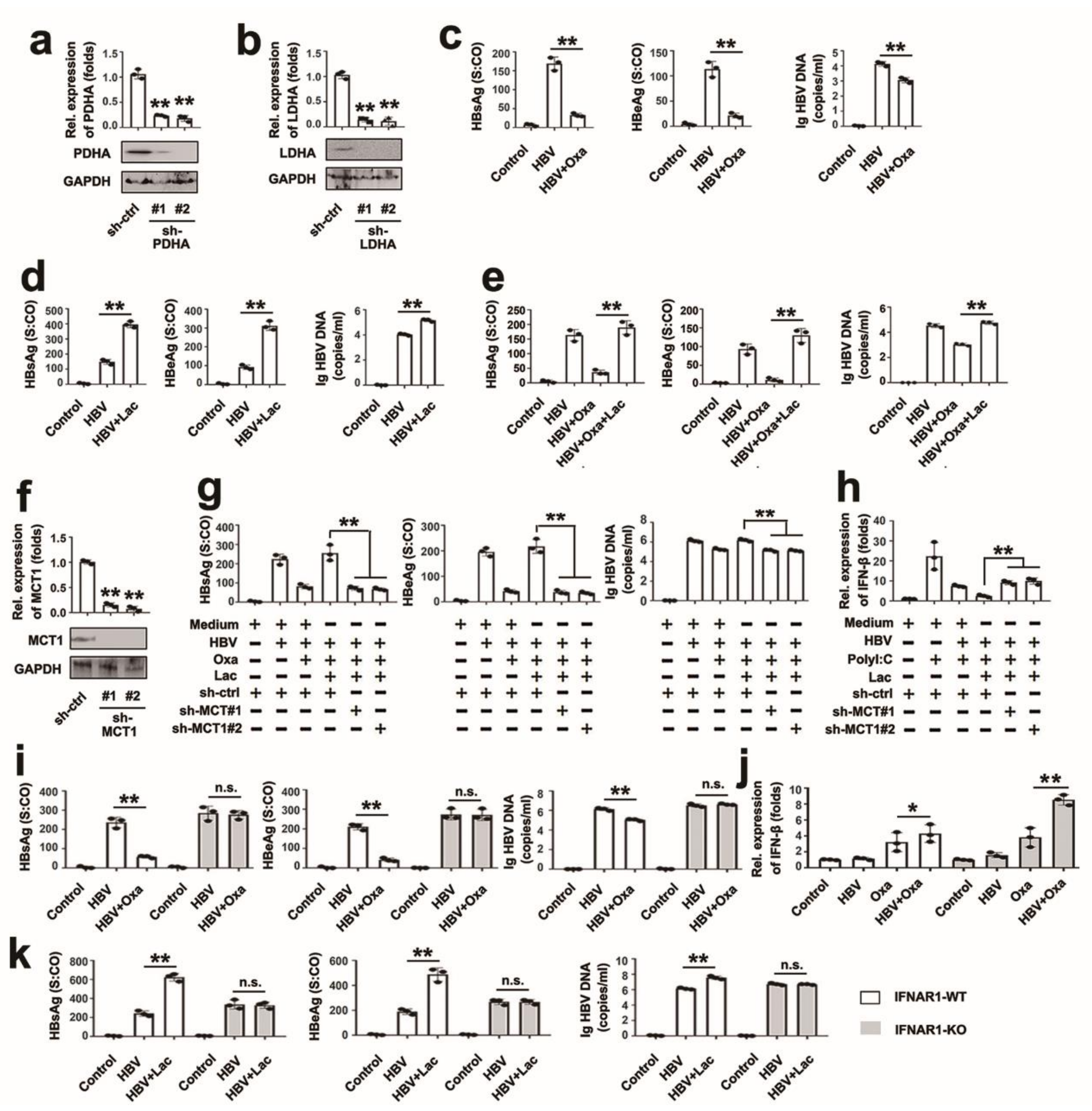

Supplementary Figure 6. HBV inhibits IFN production through LDHA-associated lactate, Related to Figure 4.

(a and b) HepG2 cells were shRNA-ctrl and shRNA-PDHAs (a) or shRNA-LDHAs (b) for 48 h prior to qPCR (upper panel) and western blot (lower panel) assays.

(c) PHHs were infected with or without HBV virions (MOI=30) for 7 d, and treated with or without sodium oxamate for 24 h. HBsAg (left panel) and HBeAg (middle panel) secreted in culture supernatants were quantified by CMIA, or the levels of HBV genomes in culture supernatants were determined using qPCR (right panels).

(d) Experiments were performed as in (c), except lactate were used.

(e) Experiments were performed as in (c), except PHHs were treated with or without sodium oxamate and lactate.

(f) Experiments were performed as in (A), except shRNA-MCT1s were used.

(g) HepG2-NTCP cells were infected with or without HBV virions (MOI=300) for 3 days. Cells were transfected the indicated shRNAs for 24 h, and treated with or without sodium oxamate and lactate for 24 h. HBsAg (left panel) and HBeAg (middle panel) secreted in culture supernatants were quantified by CMIA, or the levels of HBV genomes in culture supernatants were determined using qPCR (right panels).

(h) HepG2-NTCP cells were infected with or without HBV virions (MOI=300) for 3 days and transfected the indicated plasmids or shRNAs for 36 h. Then, cells were treated with or without lactate for 12 h, transfected with or without poly(I:C) (1 μg/mL) for 12 h, and subjected to qPCR analyses.
(i) IFNAR WT (WT) and IFNAR$^{-/-}$ cells were transfected with control vector or pHBV-1.3 (genotype D) for 36 h, and treated with or without sodium oxamate for 24 h. HBsAg (left panel) and HBeAg (middle panel) secreted in culture supernatants were quantified by CMIA, or the levels of HBV genomes in culture supernatants were determined using qPCR (right panels).
(j) Experiments were performed as in (i), except IFN-β mRNA levels were measured using qPCR.
(k) Experiments were performed as in (i), except lactate were used.
Data (a-k) represent means ± SD (Student's t-test) (**P < 0.01; *P < 0.05, n.s., not significant)

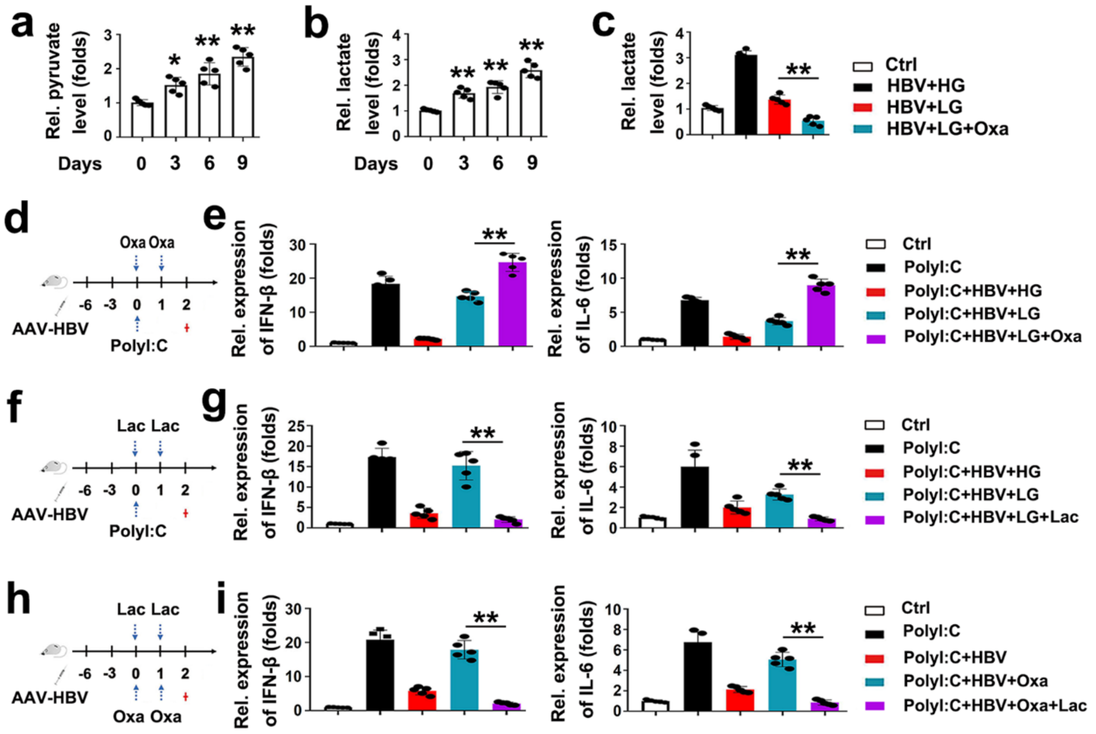

Supplementary Figure 7. HBV inhibits RLR signaling through LDHA-associated lactate *in vivo*, Related to Figure 5.

(a and b) C57BL/6 mice (n=5 for each group) received HI with 10 μg of plasmid pAAV-HBV1.2 for the indicated times prior to measuring serum pyruvate (a) and lactate (b) levels.

(c) C57BL/6 mice (n=5 for each group) received HI with 10 μg of plasmid pAAV-HBV1.2. At 6 days after HI, the mice were treated with high glucose (1.5 g/kg) or low glucose (0.2 g/kg) with or without injection of sodium oxamate (750 mg/kg) for 2 days. Serum lactate levels were measured.

(d and e) C57BL/6 mice (n = 5 for each group) received HI with 10 μg of plasmid pAAV-HBV1.2. At 6 days after HI, the mice were treated with high glucose (1.5 g/kg) or low glucose (0.2 g/kg) with or without injection of polyI:C or sodium oxamate (750 mg/kg) for 2 days (d). IFN-β (left panel) and IL-6 (right panel) mRNA levels in liver were quantified by qPCR.

(f and g) Experiments were performed as in (d and e), except sodium lactate (1 g/kg) was used.

(h and i) Experiments were performed as in (d and e), except mice were injected with both sodium lactate and sodium oxamate.

Data (a-c, e, g and i) represent means ± SEM (one-way ANOVA) (**$P < 0.01$; *$P < 0.05$, n.s., not significant).

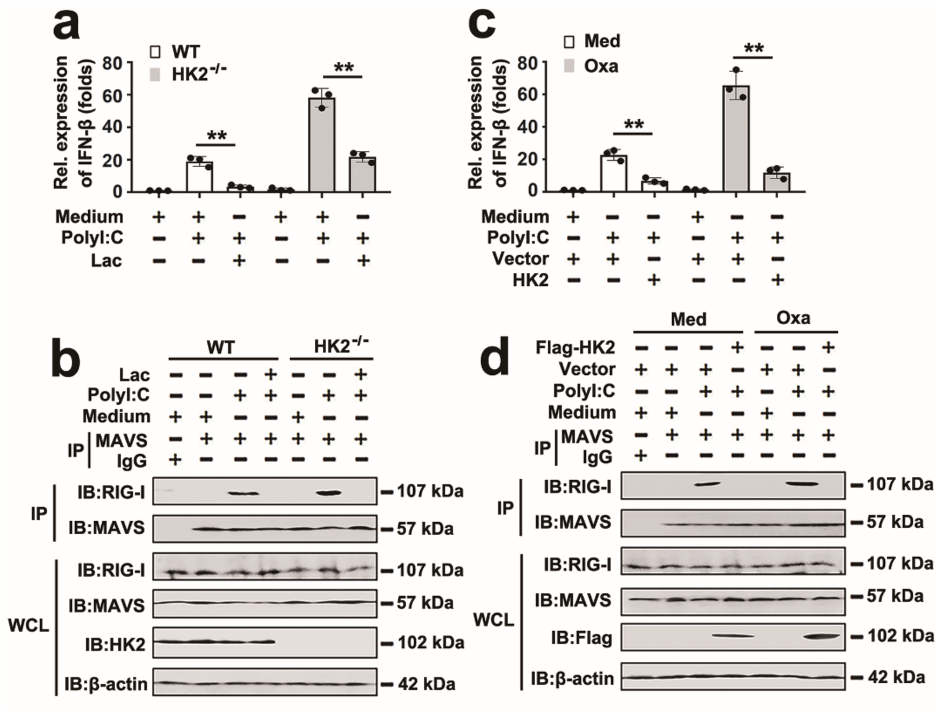

Supplementary Figure 8. HK2 and lactate inhibit MAVS activity in a cascaded manner, Related to Figure 6.

(a) HK2 WT (WT) and HK2$^{-/-}$ cells were treated with or without lactate for 12 h, and transfected with or without poly(I:C) for 12 h prior to qPCR analyses.

(b) Experiments were performed as in (a), except Co-IP and immunoblot analysis were performed with the indicated antibodies.

(c) HepG2 cells were transfected with control vector or pCMV-HK2 for 36 h and treated with or without sodium oxamate for 12 h. Then, cells were transfected with or without poly(I:C) for 12 h, and subjected to qPCR analyses for IFN-β.

(d) Experiments were performed as in (c), except Co-IP and immunoblot analysis were performed with the indicated antibodies.

Data (a and c) represent means ± SD (Student's t-test) (**P < 0.01)

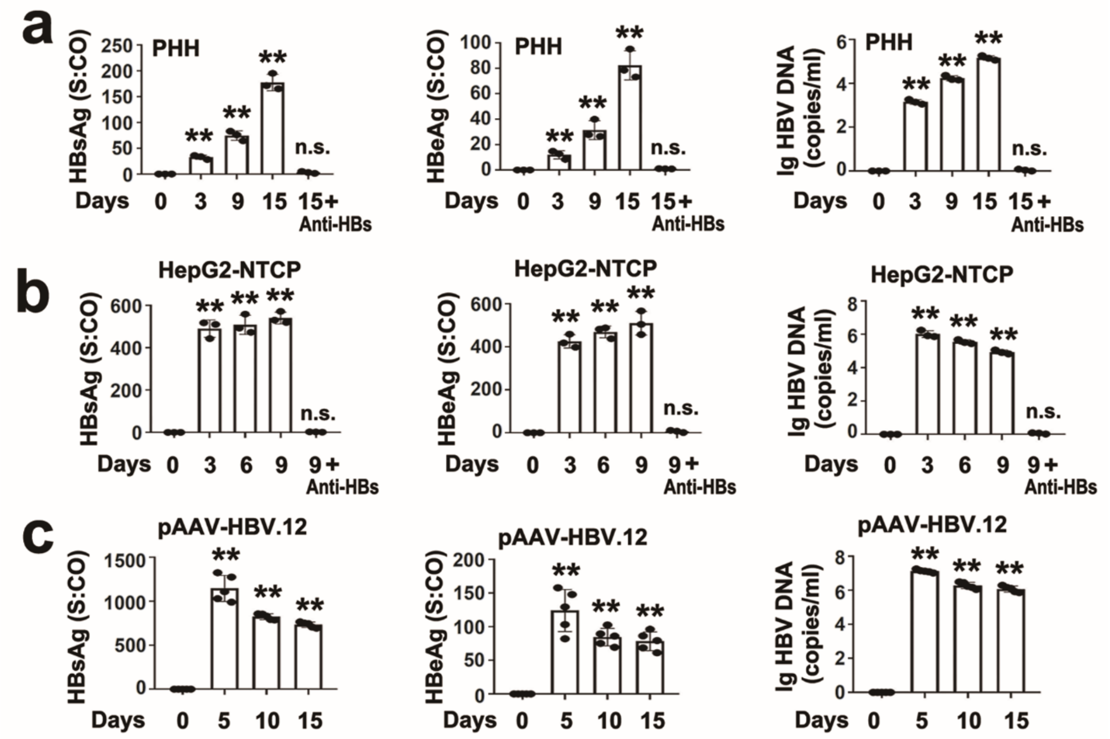

Supplementary Figure 9. Replication of HBV in PHH, HepG2-NTCP and hydrodynamic injected mice.

(a) PHHs were infected with HBV virions (MOI=30) for indicated times. HBsAg, HBeAg and HBV genomes in culture supernatants were quantified by CMIA or qPCR .

(b) HepG2-NTCP cells were infected with HBV virions (MOI=300) for indicated times. HBsAg, HBeAg and HBV genomes in culture supernatants were quantified by CMIA or qPCR .

(c) C57BL/6 mice (n=5 for each group) received HI with 10 μg of plasmid pAAV-HBV1.2 for the indicated times prior to CMIA or qPCR analyses.

Data (a and b) represent the means ± SD (Student's t-test), data (c) represent the means ± SEM (one-way ANOVA) (**P < 0.01; *P < 0.05, n.s., not significant).

Supplementary Table 1. Reagents used in this study

Reagents	Source	Identifier
TRIzol	Thermo Fisher Scientific	Cat# 15596018
2-DG	Sigma-Aldrich	Cat# D8375
DCA	Sigma-Aldrich	Cat# 347795
UK5099	Sigma-Aldrich	Cat# PZ0160
Poly(I:C)	Sigma-Aldrich	Cat# P1530
LPS	Sigma-Aldrich	Cat# L3012
HT-DNA	Sigma-Aldrich	Cat# D6898
Akti1/2	Sigma-Aldrich	Cat# 124018
Acridine orange	Sigma-Aldrich	Cat# A9231
3-MA	Sigma-Aldrich	Cat# M928
chloroquine	Sigma-Aldrich	Cat# C6628
CID1067700	Sigma-Aldrich	Cat# SML0545
LysoBeacon Green	Gene Copoeia	Cat# C048
LysoTracker Red	Thermo Fisher Scientific	Cat# L12492
EBSS	Thermo Fisher Scientific	Cat# 14,155,048
D-(+)-Glucose	Sigma-Aldrich	Cat# G7021
L(+)-Lactic acid	Sigma-Aldrich	Cat# L1750
Sodium L-lactate	Sigma-Aldrich	Cat# L7022
Sodium Oxamate	Sigma-Aldrich	Cat# O2751
D-(+)-Galactose	Sigma-Aldrich	Cat# G0750

Supplementary Table 2. Antibodies used in this study

Antibodies	Source	Dilution
HK2	Abcam (ab104836)	1:500
TOM20	Abcam (ab186735)	1:10000
HBcAg	Abcam (ab8637)	1:1000
HBsAg	Abcam (ab9193)	1:1000
SQSTM1	Abcam (ab91526)	1:5000
LC3B	Cell Signaling Technology (2775)	1:1000
LAMP1	Sigma (9091)	1:500
MAVS	Abcam (ab31334)	1:5000
Core	Abcam (ab8637)	1:1000
HA	Sigma (H6908)	1:2000
Flag	Sigma (M2)	1:2000
Myc	Abcam (ab32)	1:2000
HBx	Abcam (ab 39716)	1:1000
β-actin	Abcam (ab179467)	1:5000

Supplementary Table 3. shRNAs used in this study

Oligonucleotides	Source	Identifier
Sh-VDAC1#1	GenePharma	Cat#G08981555
Sh-VDAC1#2	GenePharma	Cat#G08981557
Sh-LDHA#1	GenePharma	Cat#G15893635
Sh-LDHA#2	GenePharma	Cat#G15893636
Sh-MCT1#1	RiboBio	Cat# stB0007914A
Sh-MCT1#2	RiboBio	Cat# stB0007914B
Sh-PDHA#1	GenePharma	Cat# G25981745
Sh-PDHA#2	GenePharma	Cat# G25981746

Supplementary Table 4. Primers used in qRT-PCR

**Gene name**	**5'primer**	**3'primer**
**IL-6 (m)**	5'-AGCCCACCAAGAACGATAGTCAA-3'	5'-TCATTTCCACGATTTCCCAGA-3'
**HBV DNA**	5'-GTTGCCCGTTTGTCCTCTAATTC -3'	5'-GGAGGGATACATAGAGGTTCCTT -3'
**IFN-β (m)**	5'- CGTGGGAGATGTCCTCAACT-3'	5'- AGATCTCTGCTCGGACCACC -3'
**IFN-β(h)**	5'-TGGGAGGCTTGAATACTGCCTCAA -3'	5'- TCCTTGGCCTTCAGGTAATGCAGA -3'
**IL-6 (h)**	5'-ACTCACCTCTTCAGAACGAATTG-3'	5'-AGCCATCTTTGGAAGGTTCAG -3'
**GAPDH (h)**	5'-GGAAGGTGAAGGTCGGAGTCA -3'	5'-CTCGCTCCTGGAAGATGGTGATGGG -3'
**β-actin (m)**	5'- GATCATTGCTCCTCCTGAGC -3'	5'- ACATCTGCTGGAAGGTGGAC -3'

Abbreviations: H, human; m, mouse.
